# Time scales and wave formation in non-linear spatial public goods games

**DOI:** 10.1371/journal.pcbi.1007361

**Published:** 2019-09-23

**Authors:** Gregory J. Kimmel, Philip Gerlee, Philipp M. Altrock

**Affiliations:** 1 Department of Integrated Mathematical Oncology, H. Lee Moffitt Cancer Center and Research Institute, Tampa, Florida, United States of America; 2 Department of Mathematical Sciences, Chalmers University of Technology, Gothenburg, Sweden; 3 Department of Mathematical Sciences, University of Gothenburg, Gothenburg, Sweden; 4 University of South Florida, Morsani College of Medicine, Tampa, Florida, United States of America; University of California Irvine, UNITED STATES

## Abstract

The co-evolutionary dynamics of competing populations can be strongly affected by frequency-dependent selection and spatial population structure. As co-evolving populations grow into a spatial domain, their initial spatial arrangement and their growth rate differences are important factors that determine the long-term outcome. We here model producer and free-rider co-evolution in the context of a diffusive public good (PG) that is produced by the producers at a cost but evokes local concentration-dependent growth benefits to all. The benefit of the PG can be non-linearly dependent on public good concentration. We consider the spatial growth dynamics of producers and free-riders in one, two and three dimensions by modeling producer cell, free-rider cell and public good densities in space, driven by the processes of birth, death and diffusion (cell movement and public good distribution). Typically, one population goes extinct, but the time-scale of this process varies with initial conditions and the growth rate functions. We establish that spatial variation is transient regardless of dimensionality, and that structured initial conditions lead to increasing times to get close to an extinction state, called *ε*-extinction time. Further, we find that uncorrelated initial spatial structures do not influence this *ε*-extinction time in comparison to a corresponding well-mixed (non-spatial) system. In order to estimate the *ε*-extinction time of either free-riders or producers we derive a slow manifold solution. For invading populations, i.e. for populations that are initially highly segregated, we observe a traveling wave, whose speed can be calculated. Our results provide quantitative predictions for the transient spatial dynamics of cooperative traits under pressure of extinction.

## Author summary

Evolutionary public good (PG) games capture the essence of production of growth-beneficial factors that are vulnerable to exploitation by free-riders who do not carry the cost of production. PGs emerge in cellular populations, for example in growing bacteria and cancer cells. We study the eco-evolutionary dynamics of a PG in populations that grow in space. In our model, PG-producer cells and free-rider cells can grow according to their own birth and death rates. Co-evolution occurs due to public good-driven surplus in the intrinsic growth rates at a cost to producers. A net growth rate-benefit to free-riders leads to the well-known tragedy of the commons in which producers go extinct. What is often omitted from discussions is the time scale on which this extinction can occur, especially in spatial populations. Here, we derive analytical estimates of the *ε*-extinction time in different spatial settings. As we do not consider a stochastic process, the *ε*-extinction time captures the time needed to approach an extinction state. We identify spatial scenarios in which extinction takes long enough such that the tragedy of the commons never occurs within a meaningful lifetime of the system. Using numerical simulations we analyze the deviations from our analytical predictions.

## Introduction

Heterogeneity and spatial patterns in population dynamics appear spontaneously in nature, on a wide range of spatial and temporal scales [1, 2, 3, 4]. Populations of reproducing individuals are not randomly dispersed, but aggregate according to climate, predatory stress, and resource levels, all of which can vary in space and time. Structures of this type are, however, not always the result of external factors, but can also arise due to interactions between individuals within the population [5]. Thus, growing cell populations can be simultaneously driven by density-dependent and frequency-dependent selection [6], and the combination of the two mechanisms can lead to novel phenomena [7, 8]. Interactions between individual organisms are often mediated by their phenotypes. In terms of reproductive success, the fitness of a certain type often depends on the frequency of other types present in the population. This frequency-dependence sets the stage for game-theoretic explanations of population dynamics, the phenotype becomes a strategy. The ecological public goods game (PGG) [3] describes a scenario in which a subpopulation releases costly factors, such as enzymes or growth factors, into the environment, where they benefit both the producers and non-producers (free-riders).

The PGG is played between producers of the public good and free-riders. Individuals either produce public good and thus ‘cooperate’, or only reap the benefits, i.e. free-ride and thus ‘defect’ [9]. This population game has been studied by considering a group of *N* players [10], in which producers contribute the good at an individual cost *κ* > 0. In the case that the benefit of the good is outweighed by the cost of production, free-riders will invade and outcompete the producers, leading to the tragedy of the commons [11] in which the overall population fitness declines as free-riders take over. This social dilemma-setting may also be observed in cancer cell growth kinetics [12, 13], in which a subset of the population produces a growth factor (e.g. testosterone in prostate, endothelial growth factor in lung cancer, and platelet-derived growth factor in glioma [14]). These situations call for explicit modeling of space, since the growth factor tends to be localized to producer cells and is transported by means of diffusion, which can have a limited range. Komarova *et. al.* discussed different mechanisms that impact the time to which we see the emergence of complex traits (e.g. the production of a public good) [15]. These mechanisms may require the accumulation of multiple individual mutations that are individually deleterious. Thus one can investigate the mechanisms of sequential hits vs. the emergence of division of labor based on the occurrence of cheaters and cooperators, with applications in biofilms, cancer and viral infections such as HIV, where the public good could also include advantageous genetic material.

In a highly relevant cancer cell setting, Zhang et. al. in [16] employed a three population Lotka-Volterra model that considered cells requiring exogenous androgen (T+), cells which can produce androgen (TP), and cells which do not require androgen (T-). They showed via mathematical modeling the utility of adaptive therapy to control tumor growth, rather than hitting the tumor with the maximum tolerated dose (MTD). In a pilot clinical trial [16], the effectiveness was verified by a large improvement in median survival. In this setting, the T+ cells act as free-riders, and the TP cells act as producers. The additional drug-resistant clone cell type T- is relevant in the presence of therapy. In our work here, we consider interactions of the nature described between the two types T+ and TP. The time for one cell type to overtake the other is of significant importance, as it is renders whether tumor control is feasible in a biologically or clinically relevant time frame. Chemotherapy and targeted therapy protocols advise the MTD, which targets to eliminate drug-sensitive cells and often selects for drug-resistance. Relapse is then caused by proliferation of drug-resistant cells [17, 18, 19]. As an alternative, adaptive therapy (AT) has recently been used successfully in cancer treatments [20]. AT introduces a variable dosing schedule to control (in theory) the diversity of the cancer, and thus its growth, without eradicating it. Proliferation of drug-sensitive cells allows for greater competition (e.g. contact inhibition, resource allocations) between cell types, which inhibits the proliferation of drug-resistant tumor cells. Clinical trials in breast [21], ovarian [22, 23] and prostate [16] have demonstrated that evolution-based AT strategies can be successfully employed, potentially indefinitely, and can be superior to standard MTD. The success of these approaches might critically depend on knowledge about the time scales of extinction of producer cells.

The type of evolutionary game, and also the spatial arrangement can determine the outcomes of population dynamics [24]. Spatial PGGs have been studied mostly in populations of fixed size, as this case resembles the essence of competition and co-evolution, e.g. at carrying capacity. PGG evolutionary dynamics in growing populations has only recently been investigated in a non-spatial setting [8]. The time to reach an equilibrium point, which we denote “*ε*-fixation time”, or “*ε*-extinction time” in the case of a monomorphic equilibrium point, may depend critically on differences in net growth rates. Cooperation between cell lines was studied under varying substrate concentrations, and it was observed that segregation occurred more readily when substrate was limited [25]. These spatial pattern formations occurred as the population moved and grew into an unoccupied domain. Once the population approaches capacity, competition should take over and the dominant clone should fixate. The experiments however focused on the behavior of the initial front type and showed that variation in outcome was due to available substrate. The timing of outcomes has not been studied in great detail so far, partly because standard tools in evolutionary game theory–such as the replicator equation–can describe homogeneously growing populations [26], but do not capture differences in net growth rates that result from frequency-dependent selection, e.g. in context of a PGG [27]. However, these time scales play an important role biologically, especially if the time to reach an equilibrium is longer than the expected lifespan of the system. Tumor growth is a typical example, where the total tumor burden might kill the patient before one cell type outcompetes the other.

We take two important steps to extend the logistic population growth model considered in [8]. First, we allow for spatial variability, which can allow for rich dynamics depending on the relative magnitude of population dispersal (cell type specific diffusion coefficients). We analyze spatial heterogeneity in up to three dimensions and show that the *ε*-extinction time can be influenced by spatial heterogeneity. By spatial heterogeneity and variability we are referring to the initial (possibly) uneven distribution of cell and public good concentrations. In particular, non-random initial conditions can cause large increases in the *ε*-extinction time. We also consider the public good function to be a nonlinear, sigmoidal function. This non-linear relationship can lead to bi-stability and potential polymorphic equilibria [27, 28, 29]. We are primarily concerned with how spatial variations impact the time it takes to reach an extinction event. To approximate the *ε*-extinction time in spatial systems, we show under what conditions the well-mixed (non-spatial) model can elucidate a decent approximation through the time it takes to travel along the “slow manifold”. In certain parameter regions, we calculate an estimate of the time spent on this manifold. Numerical simulations are often in good agreement with analytically estimated *ε*-extinction times, except when the nonlinearity is strong. In cases of highly non-linear growth rate, analytical approximations of the slow manifold become increasingly cumbersome although the non-spatial model provides an accurate estimate of the time. Highly structured situations can occur if producers and free-riders occupy mutually exclusive regions in space. In this case, we observe Fisher-like traveling wave solutions, with an interesting transition between pushed and pulled waves occurring at a critical threshold of the nonlinearity [30]. Thus, one can explore the time a traveling wave of free-riders needs to move across the entire domain. The so determined time scales of the eco-evolutionary PGG dynamics could then effectively be used to infer the underlying fitness functions that drive the co-evolutionary dynamics of producers and free-riders.

## Methods

### Ecological public good dynamics in space

Let us assume that producer cells (*U*) and free-riders (*V*) are closely related cell types experiencing the same baseline growth rate *α* and potentially different death rates *μ*_*U*_, *μ*_*V*_. Next, we assume that the public good, produced by *U* cells, has a non-decreasing effect on the growth rates, in the form of a multiplicative benefit to the growth rate. This benefit depends on the local public good or growth factor concentration (density) *G*, which is determined by the local producer cell density: *G* is produced by *U* cells, at a rate *ρ*, at a cost to their growth rate *κ*, and it is consumed by *U* and *V* cells alike at a rate *δ*. The diffusion rate of the public good is Γ_*G*_. We have neglected a decay rate of the public good based on the fact that there are molecules that can serve as public goods, which exhibit low decay rates due to binding and unbinding with cell surface proteins, which enhances persistence of these molecules in the long-term (see section 1, S1 Model Analysis).

The cells are assumed to reside and grow on a spatial domain ${\left[0,L\right]}^{n}\subset R^{n}$, where *n* = 1, 2, 3 is the dimension of the system. We assume that the domain has no-flux boundary conditions (e.g. cells cannot enter or leave the domain). We assume that growth, death and competition processes are purely local and that migration (determined by the cell type specific diffusion coefficients Γ_*U*,*V*_) is isotropic and involved only with nearest neighbors. We then obtain the following set of coupled PDEs that model the concentration of producer cells, free-rider cells, and public good in time and space:

$$\dot{U}\;=\Gamma_{U}\nabla^{2}U+\left[\lambda \left(G\right)-\kappa \right]\left[1-\left(U+V\right)\right]U-\mu_{U}U,$$ (1)

$$\dot{V}\;=\Gamma_{V}\nabla^{2}V+\lambda \left(G\right)\left[1-\left(U+V\right)\right]V-\mu_{V}V,$$ (2)

$$\dot{G}\;=\Gamma_{G}\nabla^{2}G+\rho U-\delta G\left(U+V\right).$$ (3)

Here, the respective growth rate is

$$\begin{array}{c} \lambda \left(G\right)=\alpha \frac{1+e^{\sigma }}{1+e^{\sigma -\beta G}}. \end{array}$$ (4)

A well-mixed version of this model was studied in [27]. It was shown that saddle-node bifurcations and other interesting features are in general impossible for a linear public good. Richer dynamics are possible when the good enters nonlinearly. Here, *σ* is a concentration-independent parameter, and *β* is the concentration-dependent parameter. These two parameters modulate the size of the nonlinearities in the growth rates. We can think of *β* as the per public good “unit” benefit to the growth rate. Whereas *σ* controls the maximal benefit obtainable. The ratio *σ*/*β* defines the location of an inflection point in the growth rates, which is the point that separates regions of synergistic and diminishing return, as a function of increasing growth factor *G*. In the small benefit-limit, *β* ≪ 1, we obtain a linear good λ(*G*) ≈ *α*(1 + *sGβ*), where *s* = *e*^*σ*^/(1 + *e*^*σ*^). Finally, producer cells experience a growth rate detriment in amount of the linear cost *κ*, as seen in Eq (1). All important parameters and their baseline values are summarized in Table 1. The typical cell size is on the order of micrometers. Thus, in an attempt to simulate many cells, we focused on spatial domain ranges of *L* = 0.1–10 cm. The length of time for a cell cycle is highly variable. A typical cell cycle could range from hours, to days, to weeks, and the PG-independent proliferation (growth) rate is typically (but not always) higher than the death rate [8].

**Table 1:** Dimensional parameters used in the model given by Eqs (1)–(3). The unit cc^-1^ means per cell cycle.

Parameter	Symbol	Typical ranges (values)	Reference
Producer’s diffusion coefficient	Γ_*U*_	10^−8^ − 10^−10^ cm^2^/s	[31]
Free-rider’s diffusion coefficient	Γ_*V*_	10^−8^ − 10^−10^ cm^2^/s	[31]
Public good’s diffusion coefficient	Γ_*G*_	10^−7^ − 10^−4^ cm^2^/s	[32, 33]
Cellular intrinsic growth rate	*α*	1 cc^-1^	
Producer’s death rate	*μ*_*U*_	< 1 cc^-1^	
Free-rider’s death rate	*μ*_*V*_	< 1 cc^-1^	
Public good production cost	*κ*	≪ 1 cc^-1^	[34]
Public good production rate	*ρ*	100-1000 cc^-1^	[35]
Public good consumption rate	*δ*	100-1000 cc^-1^	
Public good benefit (conc. independent)	*σ*	1-3	[27]
Public good benefit (conc. dependent)	*β*	2-6 [conc.]^-1^	[27]
Characteristic length of spatial domain	*L*	1-10 cm	

We can construct the following non-dimensional form of the spatial model. In the original model formulation we have eleven parameters and three initial conditions *U*_0_(*x*), *V*_0_(*x*), and *G*_0_(*x*). With appropriate choices we can reduce the total number of relevant parameters to nine dimensionless parameters. Although there are many choices for the set of dimensionless parameters, we choose this set to exploit the typical fact that the time scale of the dynamics for *G* are much faster than the time scales of the dynamics of *U* and *V* [8, 36]. This is motivated by the fact that smaller objects (e.g. IGF-I and II) tend to have higher diffusion rates than cells. After appropriate rescaling, we can use the dimensionless parameters of the non-dimensional system given in Table 2.

**Table 2:** Definition of non-dimensional parameters used in the model given by Eqs (5)–(7). Ranges are given as well as the typical values used throughout the text. *ε*_exit_ is used to determine the *ε*-extinction or fixation events.

Dimensionless parameter	Symbol	Identity	Range	Typical value
Producer’s diffusion coefficient	*γ*_*U*_	$\frac{\Gamma_{U}\delta }{\Gamma_{G}\alpha }$	10^−4^ − 10^2^	0.5
Free-rider’s diffusion coefficient	*γ*_*V*_	$\frac{\Gamma_{V}\delta }{\Gamma_{G}\alpha }$	10^−4^ − 10^2^	0.5
Producer (PG independent) birth rate	*a*	$1-\frac{\kappa }{\alpha }$	0.75-0.9	0.9
Producer death rate	*c*	$\frac{\mu_{U}}{\alpha }$	0-1	0.5
Ratio of free-rider to producer death rate	*r*	$\frac{\mu_{V}}{\mu_{U}}$	> 0	1.0
Ratio of cell birth rate to consumption rate	*ϵ*	$\frac{\alpha }{\delta }$	10^−3^ − 10^−2^	2 × 10^−3^
Neighborhood of a fixed point		*ε*_exit_		10^−8^

We introduce dimensionless time *τ* = *αt* and rescale growth factor concentration by the ratio of its production to consumption rates, *G* → (*ρ*/*δ*)*G*. Space is scaled via *L*_*x*_ = *L*_*y*_ = *L*_*z*_ = (Γ_*G*_/*δ*)^1/2^, which leads to non-dimensional domain lengths between 1 and 10^3^. In our notation, the “dot” then means differentiation with respect to dimensionless time *τ* (instead of *t*), and ∇ is the differential operator with respect to the rescaled spatial variables. Then we arrive at the dimensionless system

$$\dot{U}=\gamma_{U}\nabla^{2}U+\left(\lambda \left(G\right)-1+a\right)\left[1-\left(U+V\right)\right]U-cU,$$ (5)

$$\dot{V}=\gamma_{V}\nabla^{2}V+\lambda \left(G\right)\left[1-\left(U+V\right)\right]V-crV,$$ (6)

$$\epsilon \dot{G}=\nabla^{2}G+U-G\left(U+V\right).$$ (7)

Turning to a dimensionless framework allows us to more easily exploit the separation of time scales inherent in our system. For example, the public good consumption rate is typically much faster than the proliferation rate, *ϵ* ≪ 1, and thus spatial equilibration of the public good *G* occurs relatively fast. Similarly, we can immediately see from the dimensionless system that the ratio of death rates between cell types, *r*, is an important quantity that determines the fate of cooperation, especially provided the ratio of death rate to proliferation rate in producer cells, *c*, is small.

## Results

Our analysis in this manuscript focuses on spatial populations, and is based on cell type specific growth (birth), death and diffusion rates. We focus on two sub-populations: public good producers and free-riders, and we are interested in the question of how spatial variations in producer and free-rider densities affect the long-term behavior of their dynamics, in particular the time to reach a possible equilibrium configuration. The analysis of this system is not straightforward because, although producer cells bear a cost and are thus expected to go extinct, their local concentration and the resulting fluctuations in public good availability can influence the dynamics in interesting ways. Regardless of dimensionality, we show that any initial spatial variability is transient and equilibrates to a spatially homogenous solution. Therefore, a well-mixed ODE-model can be sufficient to analyze the long-term behavior of the spatial system. We construct a coupled dynamical system which models the behavior of public good producers and free-riders and the spatial distribution of public good (growth factor) in time and space. We derive slow manifold solutions which allow us to predict the time to reach an equilibrium *ε*-(fixation or extinction time) for a wide range of parameters.

### Spatial variation is transient regardless of dimensionality

What is the impact of variability in initial conditions? To address this question, we investigated the dynamics of the system governed by Eqs (1)–(3) in one, two and three dimensions in its non-dimensional form Eqs (5)–(7). The non-dimensional length used ranged from *L* = 10 − 500 for all spatial dimensions (*n* = 1, 2, 3). To solve Eqs (5)–(7) numerically, we discretized the domain into grid points. The grid points were then given initial concentrations of the amount of producer, free-rider and public good present. The distance between grid points, or the spatial step size, was chosen to be no bigger than Δ*x* = 0.5. We tested smaller grid sizes, but found no significant changes in the dynamics, only in total CPU time. We solved the PDE using a Crank-Nicolson scheme with a time step size Δ*t* = 0.01 [37]. We also tested the sensitivity of the *ε*-extinction time to different Δ*t* and found that in all cases, Δ*t* = 0.01 was sufficient. Unless specified differently, we set *r* = 1, i.e. we assumed that the two types had equal death rates. Then, simulations were used to calculate the time to reach the neighborhood of a stable fixed point, with an exit criterion based on the 1-norm distance to the stable fixed point (U*, V*) as *d*(*U*, *V*) ≔ |*U* − U*| + |*V* − V*| < *ε*_exit_, where the value *ε*_exit_ = 10^−8^ was used. If the initial condition was noisy, 100 simulations were used to generate summary statistics.

In all settings of different spatial dimension, we were interested in three types of initial conditions that define the initial cell density (amplitude) at every grid point: (1) Uniformly distributed values between 0 and 1, (2) domain wall (step function), and (3) oscillatory. To examine the stability of the more structured density distributions (2) and (3), we also tested the impact of spatial noise by introducing a random deviation of the cell density in each point in space, which was chosen no greater than 10% of the max amplitude at each grid point.

**Fig 1:**
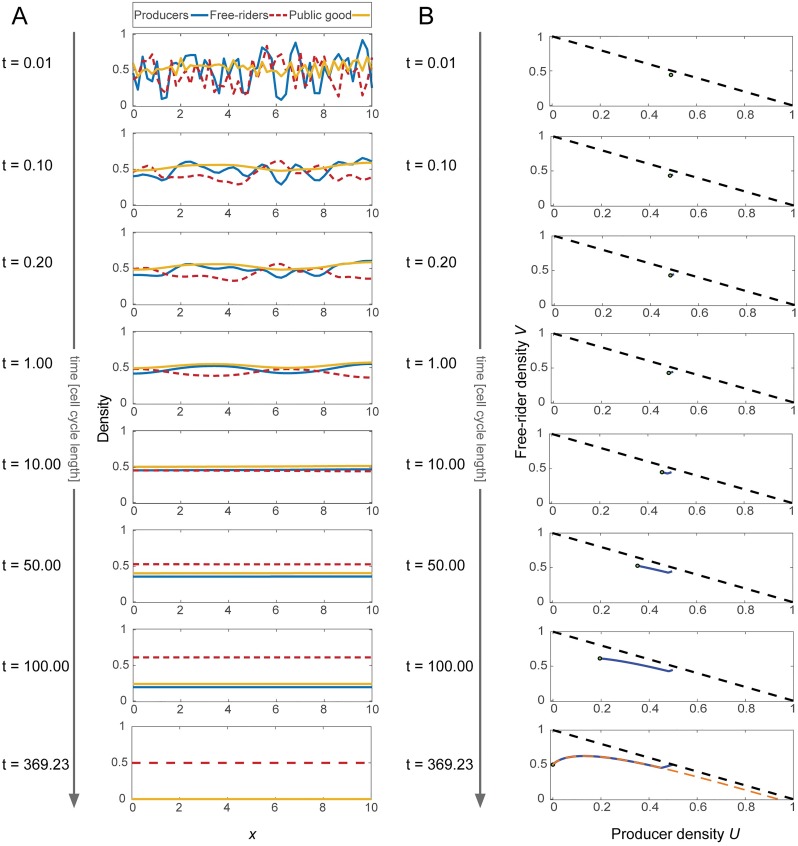
Typical 1D simulation leading to producer extinction. **A**: Snapshots of the system, represented by concentration of producer cells (*U*), free-riders (*V*) and growth factor (*G*) over time, measured in cell-cycle length (time advances top to bottom). The population game is played in 1D, the panels show the concentrations in space. **B**: Corresponding trajectory of the average number of producers and free-rider densities in their phase space (*U* = *V* on the black dashed line). Due to cell motility, the system reaches the slow manifold (orange-dashed line in bottom panel) fast, and spends most of the time traveling along the slow manifold. The slow manifold was calculated numerically from the well-mixed, ODE model. Dimensionless parameters used: *γ*_*U*_ = *γ*_*V*_ = 0.5, *a* = 0.9, *c* = 0.5, *r* = 1, *ϵ* = 2 × 10^−3^, *β* = 5, *σ* = 2.

Under the assumption of fast diffusion of cells into space, a spatial perturbation typically equilibrates along the spatial domain faster than an average cell cycle length. Fig 1 shows the temporal evolution of a typical simulation run, with a random initial condition being drawn from a standard uniform distribution on each grid point. The oscillations of initial cell densities were rapidly equilibrated during the first few cell cycles. Once the system had become roughly homogeneous, the system began to travel along the slow manifold (shown as the orange, dashed line in the final snapshot), toward free-rider fixation (producer extinction). In this example, the exit condition was met at *τ* = 489.38. The average cell concentrations are shown in the second subplots and shows the phase diagram for the average cell concentrations. The average quickly reaches the slow manifold and spends most of its time traveling along it. The final snapshot shows the slow manifold, calculated from the ODE model with a dashed, orange curve. Although the model is explicitly spatially dependent, the average cell population rapidly approaches the slow manifold of the spatially averaged cell populations. Random spatial fluctuations do not have a huge impact since on small length scales, they are smoothed rapidly (see section 6, S1 Model Analysis).

**Fig 2:**
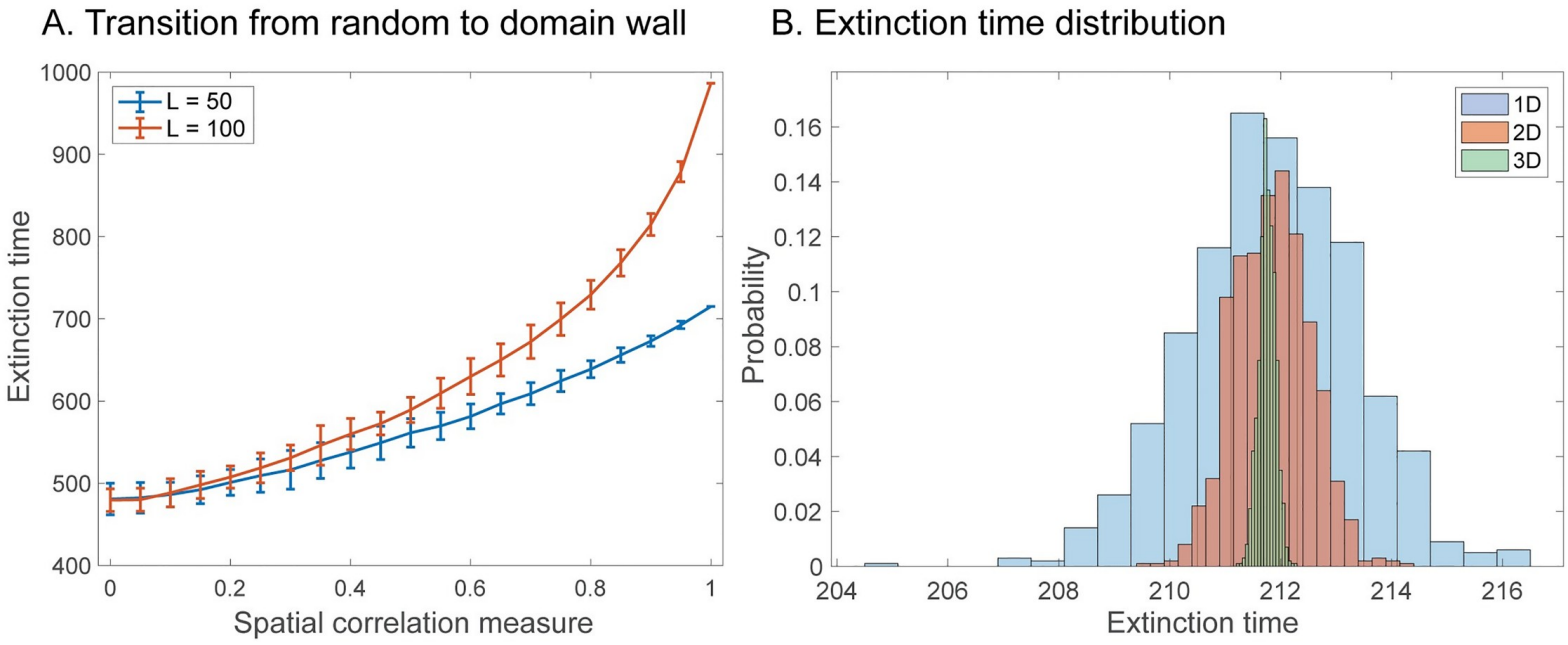
The effects of initial conditions and dimensionality. Comparison over dimensionality and random initial condition. **A** Transition from random to domain wall initial condition. We let the initial condition ${\vec{u}}_{0}=p\vec{W}+\left(1-p\right)\vec{R}$ where $\vec{W}$ is the domain wall I.C. and $\vec{R}$ is the random I.C., hence *p* can be thought of as a spatial correlation measure. Two lengths *L* = 50, 100 are shown. The simulation was done in 1D and 50 simulations were done per point. The error bars correspond to plus or minus two standard deviations. **B** Distribution of extinction times by dimension. Dimensionless parameters used *γ*_*U*_ = *γ*_*V*_ = 0.5, *a* = 0.9, *σ* = 2, *β* = 5, *c* = 0.5, *r* = 0.9, *ϵ* = 2 × 10^−3^.

All numerical solutions approached spatially homogeneous solutions consisting of only a single population under our parameter assumptions, regardless of dimensionality. We used a superposition of initial conditions defined by ${\vec{u}}_{0}=p\vec{W}+\left(1-p\right)\vec{R}$ where $\vec{W}$ (Fig 2A) is the segregated initial condition vector and $\vec{R}$ is the random initial condition vector. *p* can then be thought of as a type of spatial correlation measure with *p* = 0 “unstructured” and *p* = 1 “structured”.

Using random initial conditions, we found that the average time to *ε*-extinction was also independent of the dimensionality (Fig 2B). We note that the narrowing of the distribution of *ε*-extinction times is not related to the dimension of the system but rather to the number of grid points. This is easily seen by considering *N* random numbers drawn from a standard uniform distribution. The mean and variance are 1/2 and 1/(12*N*), respectively. The total number of grid points *N*_*i*_ where *i* is the dimension of the system was (*N*_1_, *N*_2_, *N*_3_) = (101, 441, 9261). We show that uncorrelated spatial structure evolves roughly as the mean of the initial condition (section 6, S1 Model Analysis). This is confirmed by the fact that the mean of distributions are all around 211.8 (cell cycles), and starting from the uniform state where each population is 1/2 leads to a time of 211.66 numerically. Finally, if we compare the ratio of variances of *ε*-extinction times, we expect that they should be approximately *N*_*j*_/*N*_*i*_. The results in Table 3 confirm that the distribution variability is tied to the number of grid points and not to the actual dimensionality of the system.

**Table 3:** The ratio of computed variances of extinction times and that predicted by the ratio of number of grid points.

Ratio of variances	Computed ratio	N_j_ / N_i_
1D/2D	4.8965	441/101 ≈ 4.3663
1D/3D	99.2368	9261/101 ≈ 91.6931
2D/3D	20.2669	9261/441 = 21

### Structured initial conditions stabilize the population and increase extinction times

How are *ε*-extinction times influenced by non-random initial conditions in settings of different dimensions? The impact of structured initial conditions is particularly relevant to biological processes where spatial assortment can occur in populations with limited dispersal. Therefore, we examined how the *ε*-extinction or -fixation times were affected by more coherent, non-random starting conditions.

Regardless of parameter choices, all final states are homogeneous and correspond to the stable fixed point of the non-spatial model. We thus investigated analytically the time needed to reach an equilibrium, or fixed point, using the non-spatial ODE model. To this end, we extracted an approximation which makes it possible to compare the ODE approach to the spatial PDE model. This approach allowed us to quantify the impact of spatial heterogeneity on timing to *ε*-extinction.

### The predictive power of a non-spatial approach

Numerical integration of the spatial model suggested that a non-spatial analysis could be used to determine the time scale of fixation, e.g. when public good producers go extinct. This change to a simple model system is meaningful because all final states are homogenous in space. The spatially invariant version of our dynamical system is given by

$$\dot{U}=(\lambda (G)-1+a)[1-(U+V)]U-cU,$$ (8)

$$\dot{V}=\lambda (G)[1-(U+V)]V-crV,$$ (9)

$$\epsilon \dot{G}=U-G(U+V).$$ (10)

**Fig 3:**
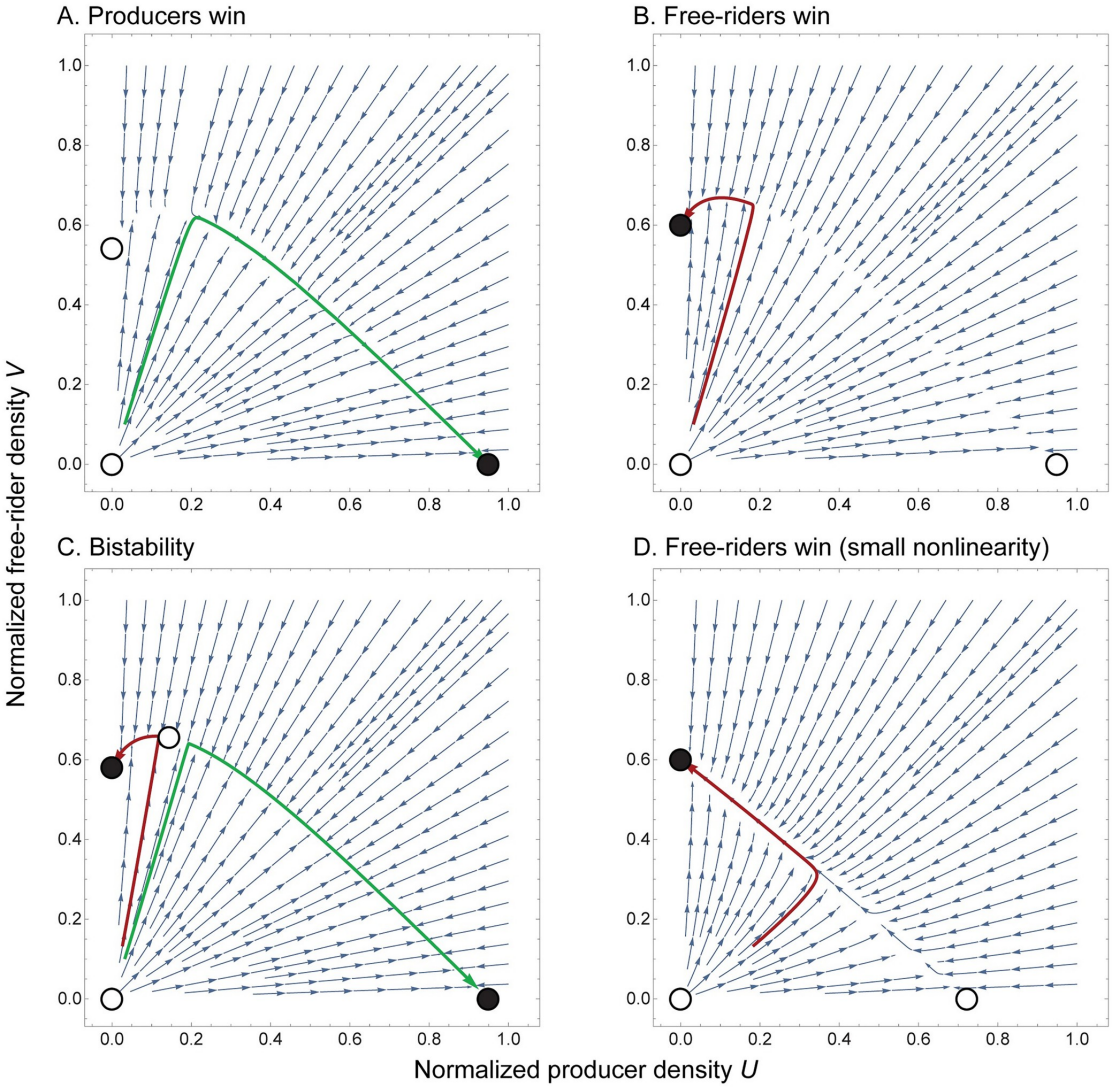
ODE phase diagrams. The fixed points are labelled by filled (stable) and hollow (unstable) circles. In all cases one observes the slow manifold which connects the fixed points. Each subplot contains trajectories (green = producers win, red = free-riders win). We also show the impact the nonlinearity has on the shape of the slow manifold. Comparing **B**, **D**, we see that the nonlinearity deviates the manifold from a straight line. **A** phase diagram where producers win. Parameter values *a* = 0.9, *β* = 5, *σ* = 2, *c* = 0.4, *r* = 1.15. **B** phase diagram where free-riders win. Parameter values *a* = 0.9, *β* = 5, *σ* = 2, *c* = 0.4, *r* = 1. **C** phase diagram with bi-stability. Parameter values *a* = 0.9, *β* = 5, *σ* = 2, *c* = 0.4, *r* = 1.05. The nonzero death rate *c* has caused the degeneracy of non-isolated fixed points to collapse, leaving behind a slow manifold along which the dynamics travel. **D** phase diagram where free-riders win. Parameter values *a* = 0.9, *β* = 0.5, *σ* = 2, *c* = 4, *r* = 1.

First, let us turn to the possible fixed points and their stability in the non-spatial setting. The system described by Eqs (8)–(10) exhibits three main steady states which exist over a wide parameter range. Fig 3 shows examples of the dynamics between these steady states in the (*U*, *V*) plane. Additionally, a sample trajectory is shown, which indicates the approach to a slow manifold that is inherent to all trajectories (if this manifold exists). We can exploit the slow manifold-dynamics to estimate the time to reach the all-free-rider state. In addition, the linear stability conditions of the steady states can be calculated (see section 1, S1 Model Analysis):

- Extinction state: (0, 0, *G*^0^) where *G*^0^ ∈ [0, 1]. This state is stable if λ(*G*^0^) < min(*cr*, 1 − *a* + *c*).
- Producers win: $\left(1-\frac{c}{\lambda (1)-1+a},0,1\right)$. This state is stable if $a>1-\lambda \left(1\right)+\max(\frac{\lambda (1)}{r},c)$.
- Free-riders win: (0, 1 − *cr*, 0). This state is stable if $\frac{1}{r}>\max\left(a,c\right)$.
- Isolated coexistence point:
  $$\begin{array}{c} (G^{*}(1-\frac{c(r-1)}{1-a}),\left(1-G^{*}\right)(1-\frac{c(r-1)}{1-a}),G^{*}), \end{array}$$ (11)
  where G* is given in Eq. (S5) (S1 Model Analysis). This state is *always* unstable.
- Non-isolated coexistence line: (G*, 1 − G*, G*). At least some finite part of this interval containing G* = 0 is stable.

It is interesting to note that in the case of equal death rates (*r* = 1), the producer-only state is necessarily unstable, since it is assumed that production of the good comes at a cost (*a* < 1). It then follows naturally that, even if we unilaterally lower the death rate of producer cell, *r* ≤ 1 the producer-only state remains unstable. Furthermore, it was shown that a nonlinear good of this particular form has at most one coexistence point [27]. In this system, this coexistence point is in fact always unstable if *c* ≠ 0 (section 1, S1 Model Analysis).

#### Slow manifold evolution

The evolution along the slow manifold is key for the characterization of the long-term dynamics of the system. Our simulations show that for uncorrelated initial conditions, after a short amount of time, the average concentration approaches a curve on which it spends most of its time (Fig 3). This curve is the slow manifold. In general, this manifold is difficult or impossible to calculate analytically and depends on the stability of the fixed points, their location and the initial condition (Fig 3A–3C). However, in certain parameter regions, we can obtain decent estimates that allow for an approximate calculation of the time to *ε*-extinction dominated by the slow manifold (for details see section 2, S1 Model Analysis). The procedure is as follows:

- Select a fixed point to investigate (e.g. free-riders).
- Find parameter region where the point is stable through linear stability analysis.
- Assume the parameters are such that one of the eigenvalues is smaller in magnitude than the others.
- The eigenvector corresponding to this eigenvalue then determines the *linear* approximation of the slow manifold near the stable fixed point.
- Higher order terms can be used to better estimate the slow manifold.
- Calculate the time it takes on the manifold to reach to exit criterion.

As mentioned above, one can include higher order terms approximating the slow manifold. By considering a series expansion, one can further refine this approximation by including successively more terms of the slow manifold. However, the algebra is often tedious, and a large number of terms may be needed, which can make anything beyond the first two orders impractical. As *β* → 0, the public good’s impact becomes effectively linear and we observe that the linear approximation to the slow manifold would perform quite well (Fig 3D).

We define the time to *ε*-extinction *T*_*U*_ and *T*_*V*_ by the amount of time it takes for producers and free-riders to go extinct, respectively. Their non-dimensional counterparts are denoted by *τ*. In numerical procedures we specify an extinction event to occur at the threshold distance from an all-*U* or all-*V* state, given by *ε*_exit_ ≪ 1. Using Eq. (S9) with Eq. (S15) (S1 Model Analysis), we obtain the estimate for the (non-dimensional) fixation time

$$\begin{array}{c} \tau_{U}=\frac{\text{ln}|\frac{U_{0}}{\varepsilon_{\text{exit}}}|}{c(1-ar)}, \end{array}$$ (12)

when *c* ≪ 1, that is the producer cells’ death rate is small compared to proliferation rate. In dimensional variables, the *ε*-extinction time of this case can be converted easily

$$\begin{array}{c} T_{U}=\frac{\ln|\frac{U_{0}}{\varepsilon_{\text{exit}}}|}{\mu_{U}-\mu_{V}(1-\frac{\kappa }{\alpha })}. \end{array}$$ (13)

For the other cases below, the corresponding expression in dimensional variables is somewhat unwieldy. In the case when producer cells’ death and proliferation rates are of comparable magnitude, 1 − *cr* ≪ 1, we can use Eq. (S9) (S1 Model Analysis) to obtain the estimation for the time to producer extinction

$$\begin{array}{c} \tau_{U}=\frac{\ln|\frac{\left(1-c\;r\pm \varepsilon_{\text{exit}}\right)\left(1-c\;r-V_{0}\right)}{\varepsilon_{\text{exit}}V_{0}}|}{1-c\;r}. \end{array}$$ (14)

The ± is needed as we can approach this from either side of the fixed point. Note we cannot use the concentration of *U* as it turns out the corresponding eigenvector contains no magnitude in that direction. There was no such issue in Eq (12) because we were free to use the producer population as the eigenvector had magnitude in that direction. Since these concentrations must be positive, we can only approach *U* = 0 from the positive direction. In contrast, we can approach *V* = 1 − *cr* from above or below. The + from above and − from below. If producers win, the *ε*-extinction time of free-riders is given by

$$\begin{array}{c} \tau_{V}=\frac{\ln|\frac{V_{0}}{\varepsilon_{\text{exit}}}|}{c(r-\frac{\lambda (1)}{\lambda (1)-1+a})}. \end{array}$$ (15)

This approximation is valid provided that the producer-only state is far from the extinction state. As the producer-only state moves towards the extinction state, the other eigenvalue −[λ(1) − 1 + *a* − *c*] determines the slow manifold and our approximation is given by

$$\begin{array}{c} \tau_{V}=\frac{\ln|\frac{V_{0}}{\varepsilon_{\text{exit}}}|}{\lambda (1)-1+a-c}. \end{array}$$ (16)

We can make some basic observations about these approximations. The choice of exit criterion, *ε*_exit_ ≪ 1, which defines when the dynamics reach an arbitrarily small neighborhood of the fixed point, grows logarithmically as ln|1/*ε*_exit_|, and not exponentially or with a power law, as one might expect. As a result, one might derive some confidence in the measured *ε*_exit_-fixation time [8] as we have defined it in this paper, especially since fixation time often refers to the mean fixation time of an individual based Markov chain model of co-evolutionary dynamics [38, 39, 40, 41, 42, 43].

**Fig 4:**
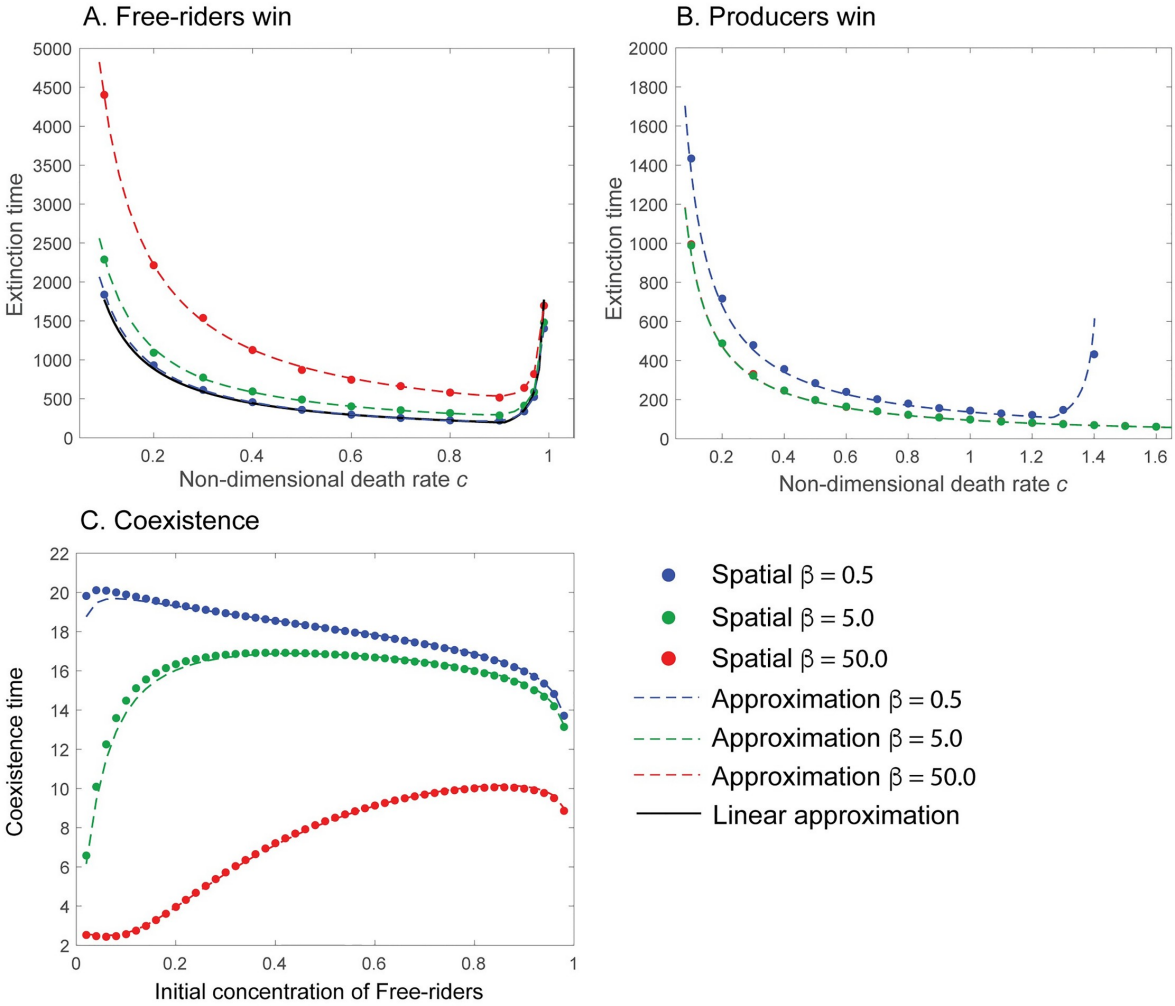
Extinction and coexistence times. (**A-B**) The time needed to reach an *ε*_exit_-radius of the stable fixed point as a function of the non-dimensional death rate *c* using 100 random initial conditions (the standard deviation was smaller than the point size): **A: Extinction time of producers**. We observe that the linear approximation (Eqs (12) and (14)) to the slow manifold suffers (black, dashed line) as it does not include the impact of *β*. The colored curves are given by using the explicit calculation of the time in the well-mixed model (Eqs (8)-(10)). Parameters used: *γ*_*U*_ = *γ*_*V*_ = 0.5, *a* = 0.9, *r* = 1, *ϵ* = 2 × 10^−3^. **B: Extinction time of free-riders**. The linear approximation (Eqs (15) and (16)) performs very well as it includes the strength of the nonlinearity *β*. Note that *β* = 5, 50 (green and red, respectively) overlap. Parameters used: *γ*_*U*_ = *γ*_*V*_ = 0.5, *a* = 0.9, *r* = 1.2, *ϵ* = 2 × 10^−3^. **C: Time to reach coexistence state/slow manifold**. Time needed to within an *ε*_exit_-radius of the coexistence manifold as a function of the initial concentration of free-riders (the initial concentration of producers is *U*_0_ = 0.01) using uniform conditions. Parameters used: *γ*_*U*_ = *γ*_*V*_ = 0.5, *a* = 0.9, *c* = 0, *r* = 1, *ϵ* = 2 × 10^−3^. All simulations were performed in the 1D system. Due to the re-scaling of the non-dimensional system, all times can be understood in units of the average cell cycle length (1/*α*).

It is interesting to note the validity of the approximation seen in Fig 4. In the case of the free-rider only state (Fig 4A), we observe that, although the slow manifold-approximation performs well (dashed colored lines), the linear approximation performs well only for small *β* (compare the black, dashed curve to the blue curve). This discrepancy makes perfect sense as the linear approximation of the slow manifold does not take into account the *form* of the public good benefit. As the benefit becomes more nonlinear (*β* increases), the linear approximation of the slow manifold to the free-rider only state will perform poorly. However, in the producer-only state (Fig 4B), we observe excellent agreement using a linear approximation to the slow manifold for all *β*. This is because the linear approximation of the slow manifold contains the form of the public good (e.g. λ(1)), and the impact of *β* as well as the functional form is retained at the first order approximation.

#### Coexistence phase

The coexistence phase can only occur when *μ*_*U*,*V*_ = 0, and it is degenerate. By degenerate we mean that the state is destroyed by any small perturbation in any relevant parameter, for example a perturbation from *c* = 0 leads to the destruction of a coexistence phase. However, one can still derive useful predictions for the time to coexistence/mutual *ε*-extinction (see section 7, S1 Model Analysis). Using Eq. (S17) with Eq. (S8) (S1 Model Analysis), we obtain an approximation of the coexistence time

$$\begin{array}{c} \tau_{\text{coexist}}=\frac{\ln|\frac{\left[U_{0}\left(1+\zeta \right)-1\right]\left[1+\varepsilon_{\text{exit}}\left(1+\zeta \right)\right]}{U_{0}\varepsilon_{\text{exit}}{\left(1+\zeta \right)}^{2}}|}{\lambda (G_{0})}, \end{array}$$ (17)

where we have defined $\zeta =V_{0}/U_{0}^{1+\xi }$, *ξ* = (1 − *a*)/λ(*G*_0_). Although *c* = 0 is an unphysical case, the approximation of the time to this curve provides an estimate for the time to the slow manifolds. To see this, observe the fact that for *c* ≪ 1, the degeneracy is broken and the non-isolated set of fixed points collapses to the boundary equilibria (all-producer or all free-rider). The time to these states is governed by the time to their respective slow manifolds. However, the time to the manifold is approximately given by the time to the coexistence point. This provides justification for why one only needs to consider the time spent on the slow manifold, as the time to the coexistence point is orders of magnitude faster. The estimation again shows good agreement across all choices of *ζ* because the growth rate-form is retained at the first order approximation (see Fig 4C).

#### Random spatial heterogeneity has little impact on extinction times

Our first-order approximations for the *ε*-extinction time provide useful insight into the parameter sensitivity of these times. For example, the *ε*-extinction time is inversely proportional to death rate and production cost, but directly proportional to the birth rate. Also, it is only logarithmically dependent on the initial concentrations and the exit threshold (proximity to the equilibrium point).

The influence of *ϵ* = *α*/*δ* (the ratio of proliferation rate over public good consumption rate) entered the approximation at higher orders and was therefore subdominant. Because of this, *ϵ* can often be assumed to be a small parameter–within a typical cell cycle, the public good produced quickly equilibrates fast. Overall, a linear approximation to the slow manifold can be used to predict *ε*-extinction and coexistence times to varying degrees of accuracy, which depends on the stability of these states (Fig 4).

### On a structured domain, the domain length can have a strong impact

To investigate the impact of the domain length, we considered uni-modal, domain wall, and random initial conditions for the concentrations of producer and free-rider cells, and for the the public good concentration. Our simulations show that the domain length did not greatly impact the *ε*-extinction time when given purely random starting conditions (see Fig 2A with *p* = 0 (uncorrelated initial conditions) and section 10, S1 Model Analysis). However, for the domain-wall and other, more structured conditions, the size of the domain influenced the fixation time substantially. Note that the invasion of free-riders into the space occupied by producers is similar to traveling waves observed in standard Fisher equations [44, 45]. We showed that total *ε*-extinction time is modified by the time it takes for this wavefront to reach the end of the unstable region, and the *ε*-extinction time can be approximated as the superposition

$$\begin{array}{c} \tau =\tau_{\text{ODE}}+\tau_{\text{wave}}+\tau_{\text{wave}\;\;\text{formation}}=\tau_{\text{ODE}}+\frac{d}{\left|\eta \right|}+\tau_{\text{wave}\;\;\text{formation}}, \end{array}$$ (18)

where *d* is the distance travelled by the Fisher wave, and |*η*| the speed of the wavefront (see section 6, S1 Model Analysis for details).

#### Free-rider invasion

In the case of an unstable producer-only state, a free-rider population initially separated will invade the producers. We consider a domain where a boundary exists between free-riders and producers. Simulations show a pushed traveling wavefront of free-riders into the producer-only region. An approximation to the speed of the wavefront is given by (see section 5, S1 Model Analysis)

$$\begin{array}{c} |\eta_{v}|=2\sqrt{c\gamma_{V}\left[\frac{\lambda \left(1\right)\left(1-r\right)+r\left(1-a\right)}{\lambda \left(1\right)+a-1}\right]}. \end{array}$$ (19)

and the total time to *ε*-extinction for *c* ≪ 1 is given by

$$\begin{array}{c} \tau_{E}\approx \frac{\ln|\frac{U_{0}}{\varepsilon_{\text{exit}}}|}{c(1-ar)}+\frac{d}{2}\sqrt{\frac{\lambda (1)+a-1}{c\gamma_{V}\left[\lambda \left(1\right)\left(1-r\right)+r\left(1-a\right)\right]}}. \end{array}$$ (20)

**Fig 5:**
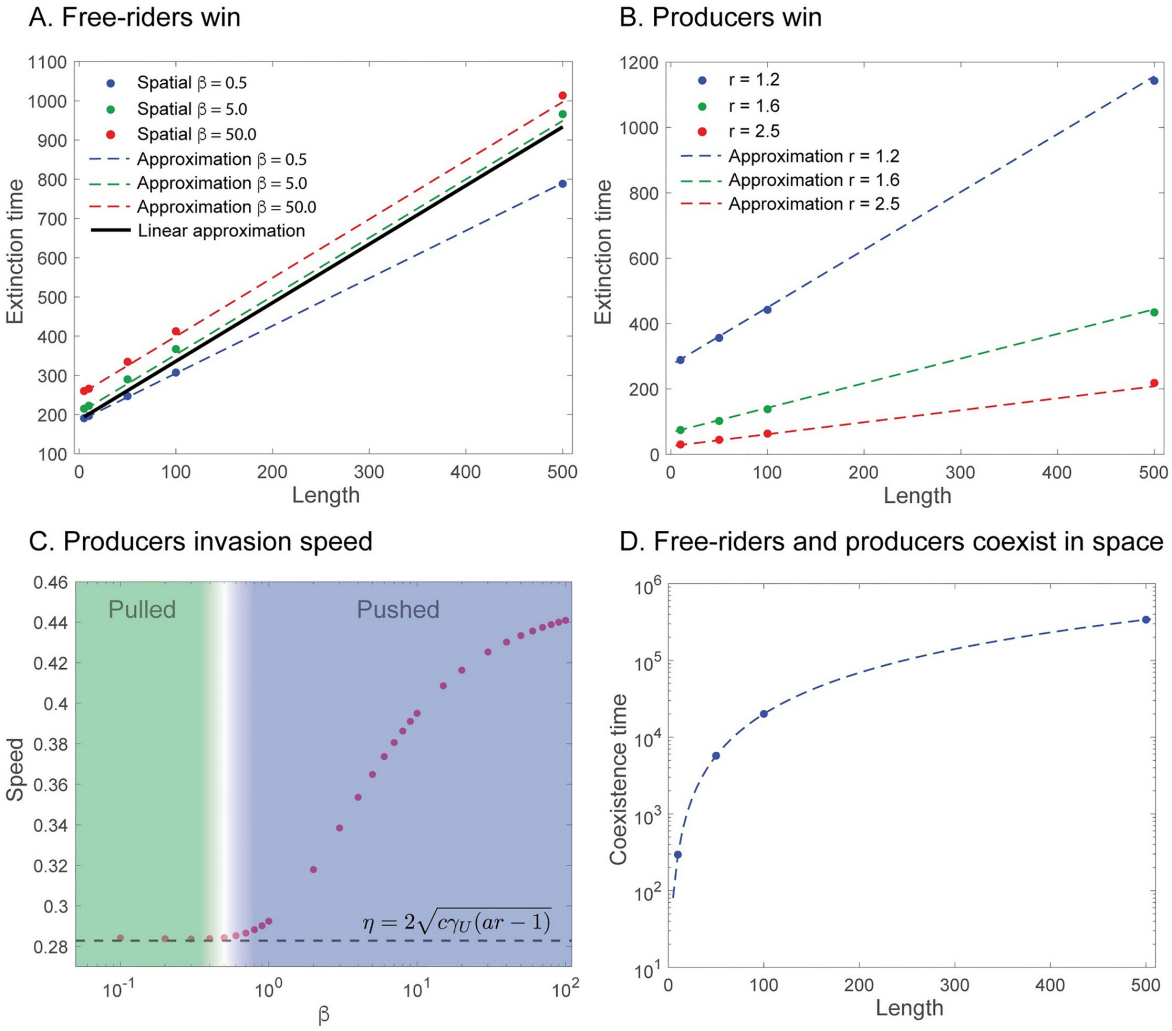
Extinction times increase linearly with length, and coexistence times increase quadratically with length. **A**: Producer extinction time vs. length of the domain. theoretical lines were obtained using Eq (20) (black, blue), and the red and green lines were obtained using the actual time of the slow manifold from the well-mixed model Eqs ((8)–(10)), with the wave front speed in Eq (19). Parameters used: *a* = 0.9, *c* = 0.5, *σ* = 2.0, *r* = 0.9, *γ*_*U*_ = *γ*_*V*_ = 0.5, *ϵ* = 2 × 10^−3^. **B**: free-rider extinction time vs. length of the domain. theoretical lines were obtained using Eq (22). Parameters used: *a* = 0.9, *c* = 0.5, *σ* = 2, *β* = 0.5, *γ*_*U*_ = *γ*_*V*_ = 0.5, *ϵ* = 2 × 10^−3^. **C**: Analysis of the transition from pulled to pushed fronts. This was only observed when the producer invades. As *β* increases, we shift from the pulled velocity predicted by linear theory. The transition occurs in the colorless zone. Parameters used: *a* = 0.9, *c* = 0.5, *r* = 1.2, *σ* = 2, *γ*_*U*_ = *γ*_*V*_ = 0.5, *ϵ* = 2 × 10^−3^. **D**: Time to reach coexistence state or time to slow manifold with *c* = 0. Dashed line obtained from Eq. (S43) (S1 Model Analysis). Parameters used: *a* = 0.9, *σ* = 2, *β* = 0.5, 5, 50, *γ*_*U*_ = *γ*_*V*_ = 0.5, *ϵ* = 2 × 10^−3^. The times to coexistence for different *β* values deviate by less than 0.1 cell cycles.

Note that this approximation is valid for *c* ≪ 1. In the case of *c* ≈ 1 we would replace the first term of the approximation (20) by the right hand side of Eq (14). We tested Eq (20) against different parameter values (see Fig 5A) and found good agreement with the prediction for *β* < 1. The poorer agreement comes from the well-mixed slow-manifold approximation performing poorly with higher *β* (Fig 4A). The time added to the ODE prediction can be described by the amount of time needed for the free-rider wavefront to travel the distance necessary to cover the entire finite domain before it takes over.

#### Producers invasion

In the case of an unstable free-rider-only state, a spatially separated producer population will invade the free-riders. Unlike the free-rider invasion, the mathematical description of this producer invasion is more complicated. This is mostly due to the impact of *r* (the ratio of free-rider to producer death rate) on the location of the free-rider-only state. Suppose we are in a region in parameter space where the free-rider-only state is unstable. If this state is in the region (0,1), we can proceed as we did in the previous section. However if 1 − *cr* < 0, which is biologically infeasible, the wavefront would travel from the mass extinction state, rather than the free-rider-only state as before. This is reflected in the different wave speeds obtained below. Thus, an approximation to the speed of the wavefront is given by (see section 5, S1 Model Analysis)

$$\begin{array}{c} |\eta_{u}|=2\{\begin{array}{cc} \sqrt{c\gamma_{U}\left(ar-1\right)} & \text{if}\;1-cr>0,\\ \sqrt{\gamma_{U}\left[\lambda \left(G^{0}\right)+a-1-c\right]} & \text{else}. \end{array} \end{array}$$ (21)

and the total time to *ε*-extinction for *c* ≪ 1 is given by

$$\begin{array}{c} \tau_{E}\approx \frac{\lambda (1)-1+a}{c[\lambda (1)(r-1)-r(1-a)]}\ln|\frac{V_{0}}{\varepsilon_{\text{exit}}}|+\frac{d}{|\eta_{u}|}. \end{array}$$ (22)

#### Pulled vs. pushed waves

It is often the case that the speed of a wavefront is not impacted by the nonlinear factors of the growth rates. Traveling waves obeying this property are said to be “pulled” waves. For large *β*, the calculated speed is *faster* than predicted by (pulled) linear theory, which suggests that there is a transition from pulled waves to pushed waves around some value of *β* < 1. Pushed waves, in general depend, on nonlinearities of the system and are more complicated to calculate [46]. However, for small *β* we observe good agreement with Eq (22), see Fig 5B, as the wave front is pulled, and linear. The breakdown of this approximation was seen only with producer invasions, shown in Fig 5C. Here, we see that the linear speed predicted increasingly under predicts actual wave speed observed in the pushed phase.

The analytical speed we calculated is the asymptotic value approached as *t* → ∞. However, since our domain is finite, calculating the speed can be tricky as it involves tracking a part of the wave numerically and calculating its speed. In addition to the wave propagation time, there is a wave formation time, which can influence the time observed. Furthermore, grid size issues introduce numerical error. To circumvent these issues and obtain the results shown in Fig 5C, we note that the total time was given by Eq (18). If we consider the time it takes to *ε*-extinction for *L* = *L*_1_ and *L* = *L*_2_ of a wave that needs to travel half the respective domain (recall *L* is the non-dimensional length of the system), then we can approximate the wave front speed *η* via the relation

$$\begin{array}{c} \eta =\frac{L_{2}-L_{1}}{2(\tau_{2}-\tau_{1})}. \end{array}$$ (23)

This result was used to compute the points in Fig 5C.

#### Diffusion times are relevant only in the absence of cell death

The special case of vanishing cellular death rates, *c* = 0, revealed the possibility of coexistence of producers and free-riders along a one-dimensional subset of the state space. In this context, it is interesting to examine the limit as *c* → 0 in an initially highly structured population, where producer and free-rider cells are segregated at time 0. In this case, the wavefront speed, e.g. of an expanding free-rider population, tends to 0. A vanishingly slow wavefront would imply that the *ε*-extinction time tends to infinity. The traveling wave is no longer the mechanism that governs equilibration. Indeed, the diffusion time that governs cellular dispersion becomes relevant. Scaling implies that the characteristic time for diffusion is *τ*_diffusion_ ∼ *L*^2^/*D*, where *D* is the diffusion coefficient of both types of cell. For *c* > 0, the wavefront should move faster than diffusion, and so this type of scaling with the diffusion constant is not seen for finite cell death rates.

Cellular diffusion should be the driving factor in long time scenarios as *c* → 0. To test this, we considered *c* = 0 and calculated the time to homogeneity. An analytical expression, Eq. (S43), for this time was calculated (section 7, S1 Model Analysis), which scales with the square of the system length. The comparison between domain length and the time scale to reach diffusion-driven coexistence in this special case of diffusion dominated *ε*-extinction is shown in Fig 5D. We would expect that this time should not be dependent on the shape of the nonlinear good because the approach to the state *U* + *V* = 1 is rapid compared to the time scale for full equilibration. Once we are near this line, the impact of the good vanishes as we are near capacity. We tested this assumption with increasingly non-linear growth rates, *β* = 0.5, 5, 50, and found no differences in coexistence times. For example, a domain length *L* = 20 had coexistence times, 1065.03, 1065.14 and 1065.16 for *β* = 0.5, 5, 50, respectively.

## Discussion

Here we considered a spatial nonlinear public goods population game model in its deterministic form. We have investigated the impact of spatial arrangement of public good producer cells and free-rider cells on the temporal scales of extinction or coexistence during the co-evolution of these populations on a finite spatial domain. The model typically exhibits fixation of either producers of the public good or free-riders, which critically depends on the frequency-dependent birth and death rates, and on properties of the public good itself, such as the cost of production. While the cost to benefit ratio plays a part in this, the overall dependencies can be more involved. Our analysis has shown that structured (correlated) initial conditions have a large impact on the predicted (*ε*) time to fixation.

The dynamics of unstructured (random) initial conditions can be captured by a non-spatial approach, for which *ε*-extinction times can be calculated analytically. In certain parameter regions, an approximation to this *ε*-extinction time can be calculated to decent accuracy. However, the process is cumbersome and when the influence of the public good is strongly nonlinear (*β* ≫ 1), the approximation requires a large number of terms to properly capture the shape of the slow manifold that dominates the time scales. The behavior of the *ε*-extinction time as a function of the death rates (Fig 4A and 4B) shows a minimum time to *ε*-fixation time. The reason for this can be understood with two observations. First, we begin with small death rate *c* ≪ 1. As this rate increases, both cell-types exhibit faster death rates and so we expect the time to *ε*-fixation to go down. However at some point, we observe the reverse, increasing the death rate is leading to an increasingly long time to reach the free-rider only state. The culprit is the death rate has become so high, that the fixed point is now “close” to the extinction state, which is a repeller. This state acts to “slow” the flow towards this point. For the example in Fig 4A, we note that as *c* → 1, the free-rider only state: 1 − *cr* approaches 0. This is a transcritical bifurcation, and the divergence in time to reach the fixed point is well known [47].

For structured initial conditions, e.g. a domain wall, one type takes over the other with time that increases linearly with the size of the spatial domain. Though this is expected, it is surprising that this time is not solely dependent on the time it takes for the wave to reach the edge of the domain. Rather, the total time depends on a linear superposition of the wavefront time, and the time for the wavefront to equilibrate. We have also shown that the linear Fisher theory that predicts the wave speed is inaccurate for increasing nonlinearity (large *β*), similar to the breakdown of the linear manifold approximation of the slow manifold. In this case, there exists a transition at a critical *β*_*c*_, which could be a function of all other relevant parameters that determine whether the wave is pushed or pulled. To find this critical value could be an exciting avenue of future analytical and computational work.

Our numerical simulations show that all spatial inhomogeneities are ultimately removed, but are not insignificant in regards to the time it takes to reach spatial homogeneity. Our results also highlight a point often ignored in the evolutionary dynamics literature, which typically focuses on the evolutionary stable states (ESS) and focuses less on the temporal dynamics of selection. Similar tendencies are apparent in the wider field of the study of ecological systems, where transient behavior has often been secondary to determining long-term stable states [48]. Our analysis shows that both population dynamical parameters, such as death rate, the initial condition, and the spatial extent of the population influence the time it takes to reach the ESS. These results are particularly relevant to cancer, where public goods might be a common feature of tumor-ecological stability, for example as seen by the evolution of autocrine growth factor production [13]. The time to the end of the game may also be quite long, perhaps greater than the lifetime of the patient.

We also investigated the possibility of diffusion-driven pattern formations via a Turing bifurcation. A typical requirement is a large difference in the relative magnitude of diffusion coefficients. We tested different scenarios, but we did not observe any pattern formations, all solutions approached homogeneity. Furthermore, we show that Turing bifurcations are not possible in this system (section 8, S1 Model Analysis). This is in contrast to other work, where heterogeneous spatial solutions and chaos were observed [3, 4].

In summary, we have considered the spatial growth dynamics of producer and free-riders, determined by a diffusible nonlinear public good, in one, two and three dimensions. Extracting a slow manifold solution, we obtained a good estimate for the time to *ε*-extinction of a cell type. For invading populations, i.e. for initially highly segregated sub-populations, we observed a traveling wave solution. We calculated an estimate of the wavefront speed and showed that the total time is given by the superposition of the traveling wave speed plus the time the well-mixed (ODE) solution needs to equilibrate to the average value of the wave profile. These were in excellent agreement with simulations provided that the nonlinearity was not too strong. The culprit was the strength of the nonlinearity *β*. When this was large, the wave transitioned from pulled to pushed. Our spatial model can be used to generalize the tumor ecological dynamics presented in [16], which was used to assess adaptive anti-cancer strategies assuming a well-mixed population. Our spatial considerations can help refine such models and provide more accurate predictions, which could reveal critical new information with regard to the time scales of population transformations.

## Supporting information

S1 Model Analysis Linear stability analysis, calculation of *ε*-extinction times, slow manifold and traveling wave speeds are conducted. We give a proof demonstrating how the wave front and well-mixed model times combine. We present comparisons of 1D-plane waves in higher dimensions (Table A). We provide snapshots of two different traveling wave solutions and show how the approach to the steady state approaches that of the well-mixed model (Figure A). We investigated uncorrelated (random) I.C. systematically with Moran I’s statistic and demonstrated that these conditions led to no change in the *ε*-extinction times (Table B).
(PDF)
