# Supplementary material for: Time scales and wave formation in non-linear spatial public goods games
Source: PLoS Comput Biol. 2019 Sep 23;15(9):e1007361. doi: 10.1371/journal.pcbi.1007361 (PMC6776369; doi:10.1371/journal.pcbi.1007361)
Supplement: S1 Model Analysis — We give a proof demonstrating how the wave front and well-mixed model times combine. We present comparisons of 1D-plane waves in higher dimensions (Table A). We provide snapshots of two different traveling wave solutions and show how the approach to the steady state approaches that of the well-mixed model (Figure A). We investigated uncorrelated (random) I.C. systematically with Moran I’s statistic and demonstrated that these conditions led to no change in the ε-extinction times (Table B). (PDF) [file pcbi.1007361.s001.pdf]

# Time scales and wave formation in non-linear spatial public goods games: Supplementary Information

Gregory J. Kimmel[1], Philip Gerlee[2,3], Philipp M. Altrock[1,4]*

**1** Department of Integrated Mathematical Oncology, Moffitt Cancer Center and Research Institute, Tampa, FL, United States of America
**2** Department of Mathematical Sciences, Chalmers University of Technology, Gothenburg, Sweden
**3** Department of Mathematical Sciences, University of Gothenburg, Gothenburg, Sweden
**4** University of South Florida, Morsani College of Medicine, Tampa, FL, United States of America
***** Email: philipp.altrock@moffitt.org

## Contents

**1 Steady states and linear stability analysis** 2

**2 Calculating $\varepsilon$-extinction times** 3

**3 Slow manifold calculation** 4

**4 Calculating time to slow manifold/coexistence times** 5

**5 Calculation of wavefront speed in 1D** 5
5.1 Free-rider invasion . . . 5
5.2 Producer invasion . . . 6
5.3 Wavefront speed in higher dimensions . . . 6

**6 Calculating $\varepsilon$-extinction times using a traveling wave ansatz** 7

**7 Calculation of time to coexistence in the absence of cell death** 9

**8 Ruling out pattern formations** 10

**9 Different initial conditions converge to the homogeneous solution** 10

**10 Uncorrelated initial conditions remove domain-length dependence** 12

# 1 Steady states and linear stability analysis

We begin with Eq. (3) from the main text, which describe the dynamics of the densities of public good (PG) producer cells, $U$, free rider cells, $V$, and the public good/growth factor itself, $G$, all of which are functions that depend on space and time. Our choice to neglect a decay rate for the public good $\mu_G$ is based on the following observations. First, there are molecules that can serve as public goods, which exhibit low decay rates due to binding and unbinding with cell surface proteins, which enhances persistence of these molecules in the long-term. For example, IGF-II (used by Archetti et al., [1]) was found to exist at low concentrations for an extended period of time in the bounded state [2]. As a first approximation, for these molecules, one can assume that the removal of IGF-II from the system is due to absorption/consumption. It is entirely reasonable that the stability condition depends on the location of $G^0$, as can be easily verified by analyzing the linear stability analysis for $(0, 0, G^0)$. Furthermore, $\lambda(G^0) \geq 1$, and we could observe then that our condition is to simply require that all monomorphic states need to be unstable. As the reviewer points out, a finite molecule-lifetime would collapse this condition to the standard fixed point (0,0,0), via the introduction of an additional (small) decay rate of the form $-\delta G$. However, this makes very little qualitative change in all other states, and aside from the collapse of the line $(0, 0, G^0)$ to $(0, 0, 0)$, no quantitative change would be observed. For example, in the free-rider only state, the location of the fixed point is unchanged at $(0, 1 - cr, 0)$, the only eigenvalue modified would be $-(1 - cr)/\epsilon \to -(1 - cr + \delta)/\epsilon$. For small public good decay rate $\delta$, this effect will be negligible. Additionally, using the fast-time scale implied by the faster diffusion constant of the PG, one could argue that the impact of this eigenvalue is damped incredibly quickly as it is $O(1/\epsilon)$, where $\epsilon = \alpha/\delta$ is the ratio of cellular growth rate to consumption rate. The producer-only state gains another solution with the additional term. However, this turns out to be a small change as this solution is necessarily negative (unphysical). The physical producer-only state solution maintains stability conditions that are based on the same eigenvalues, but with the location of the state slightly shifted due to the additional (small) $\delta$-term.

In non-dimensional form, these partial differential equations read

$$\dot{U} = \gamma_U \nabla^2 U + U(\lambda(G) - 1 + a)[1 - (U + V)] - cU, \tag{S1}$$

$$\dot{V} = \gamma_V \nabla^2 V + V\lambda(G)[1 - (U + V)] - crV, \tag{S2}$$

$$\epsilon\dot{G} = \nabla^2 G + U - G(U + V). \tag{S3}$$

The boundary equilibria can be read off by setting either $U$ or $V$ to zero. We obtain

- Extinction state: $(0, 0, G^0)$ where $G^0 \in [0, 1]$. This state is stable if $\lambda(G^0) < \min(cr, 1 - a + c)$.
- Producers win: $\left(1 - \frac{c}{\lambda(1)-1+a}, 0, 1\right)$. This state is stable if $a > 1 - \lambda(1) + \max\left(\frac{\lambda(1)}{r}, c\right)$.
- Free-riders win: $(0, 1 - cr, 0)$. This state is stable if $\frac{1}{r} > \max(a, c)$.
- Coexistence: $(U^*, V^*, G^*)$, for values see the below expression. This state is *always* unstable.

We know that at most one coexistence point can exist and that the point satisfies the relation [3]

$$\lambda(G) = \frac{r(1 - a)}{r - 1}. \tag{S4}$$

We can immediately see the unsurprising fact that for coexistence, $r > 1$. That is, we require $\mu_U < \mu_V$. In our case, we can explicitly solve for $G$ by inserting our form for the nonlinear public good and we obtain

$$G^* = \frac{1}{\beta}\left[\sigma - \ln\left(\frac{(1 + e^\sigma)(r - 1)}{r(1 - a)} - 1\right)\right]. \tag{S5}$$

Finally, we can use this to solve for $U, V$ by noting that $G = U/(U + V)$ in the coexistence state. Hence we obtain

$$(U^*, V^*, G^*) = \left(G^*\left(1 - \frac{c(r - 1)}{1 - a}\right), (1 - G^*)\left(1 - \frac{c(r - 1)}{1 - a}\right), G^*\right). \tag{S6}$$

To analyze each steady state, we need the Jacobian of the spatially-homogeneous version of Eq. (S1)-(S3).

$$J = \begin{bmatrix} (1-2U-V)[\lambda(G)-1+a]-c & U[1-a\lambda(G)] & U(1-U-V)\lambda'(G) \\ -V\lambda(G) & (1-U-2V)\lambda(G)-cr & V(1-U-V)\lambda'(G) \\ \dfrac{1-G}{\epsilon} & -\dfrac{G}{\epsilon} & -\dfrac{U+V}{\epsilon} \end{bmatrix}$$

If free-riders win the game, we obtain the eigenvalues $cr-1,\ c(ar-1),\ \frac{cr-1}{\epsilon}$. This state is stable provided that $\frac{1}{r} > \max(a,c)$. If producers win the game, we obtain the eigenvalues $1-a+c-\lambda(1),\ c\left(\frac{\lambda(1)}{\lambda(1)-1+a}-r\right),\ \frac{1-a+c-\lambda(1)}{\epsilon(\lambda(1)-1+a)}$. This state is stable provided that $\lambda(1)-1+a > \max\left(\frac{\lambda(1)}{r},c\right)$. The stability of the coexistence state involves a very complicated expression relating all the biological parameters. To gain more insight, we look at the reduced Jacobian obtained by setting $G^*=U^*/(U^*+V^*)$. Analyzing the trace and determinant of the resulting 2x2 matrix shows that in the limit as $\epsilon\to 0$, the coexistence state is always unstable.

A special case is given by $c=0$ with $U^*+V^*=1$ and $G^*=U^*$. The Jacobian simplifies considerably and we obtain eigenvalues $-\frac{1}{\epsilon},\ 0,\ G^*(1-a)-\lambda(G^*)$. Hence the coexistence state is stable if $G^*(1-a)<\lambda(G^*)$. This case is clearly degenerate in the sense that we obtain a non-isolated set of fixed points. It is clear that $\lim_{G^*\to 0}\lambda(G^*)-G^*(1-a)\to 1$ and so there exists at least some portion of the non-isolated set which is stable when $c=0$.

## 2 Calculating $\varepsilon$-extinction times

Consider a general system $\dot{x}=A(x)$ where $A(x)$ can be a nonlinear function of $x$ and $A,x\in\mathbb{R}^N$ with fixed point $x^*$ (e.g. $A(x^*)=0$). We expand near the fixed point $\varepsilon z = x-x^*$ with $\varepsilon \ll 1$ (note this is just a general small parameter, and is not the same as $\epsilon=\alpha/\delta$). This leads to

$$\dot{z}=\sum_{p=1}\frac{\varepsilon^{p-1}}{p!}\left(\sum_{n=1}^{N} z_n\frac{\partial}{\partial x_n}\right)^p A(x^*).$$

At first order, we have $\dot{z}=\sum_{n=1}^{N}\frac{\partial A(x^*)}{\partial x_n}z_n$ which is simply the linearized equation $\dot{z}=Jz$ where the components of the Jacobian were given. Assume the point $x^*$ is linearly stable and that there exists a largest eigenvalue (closest to 0) $\lambda^{(i)}>\lambda^{(j)}$ for all $j\neq i$ with corresponding eigenvector $u^{(i)}$. Then as $t\to\infty$, we expect $z(t)\to cu^{(i)}e^{\lambda^{(i)}t}$. To calculate an approximation to the slow manifold, we can without loss of generality write the eigenvector as $(1,a_2,a_3,\ldots,a_N)$ where $a_i\in\mathbb{C}$. This eigenvector approximates the direction of the slow manifold. This leads to

$$\dot{z}_1=\sum_{p=1}\frac{\varepsilon^{p-1}z_1^p}{p!}\left(\sum_{n=1}^{N}a_n\frac{\partial A(x^*)}{\partial x_n}\right)^p=\lambda^{(i)}z_1+\sum_{p=2}\frac{\varepsilon^{p-1}z_1^p}{p!}\left(\sum_{n=1}^{N}a_n\frac{\partial A_1(x^*)}{\partial x_n}\right)^p.$$

One can improve the approximation by including higher order approximations of the slow manifold. However, for our purposes, we only use the linear approximation. We also note that any higher order corrections will only enter for order $p\geq 2$. Hence an approximation to the dynamics on a 1D slow manifold is given by

$$\dot{U}=\sum_{n=1}^{N}a_nU^n. \tag{S7}$$

For simplicity, we suppose that all terms $n\geq 3$ are subdominant and can be neglected. The higher order ones are more complicated, but doable if necessary. We assume the initial value $U_0$ and we can approach the fixed point $U^*=0$ (recall that we shifted so that the fixed point is at the origin) exit criterion radius $\varepsilon_{\text{exit}}$ from either side.

therefore the time to enter the $\varepsilon_{\text{exit}}$-radius of the fixed point is given by

$$\begin{aligned} T_f &= \int_{U_0}^{U^*\pm\varepsilon_{\text{exit}}} \frac{dU}{a_1 U + a_2 U^2} \\ &= \int_{U_0}^{U^*\pm\varepsilon_{\text{exit}}} \frac{dU}{U(a_1 + a_2 U)} \\ &= \int_{U_0}^{U^*\pm\varepsilon_{\text{exit}}} \frac{1}{a_1 U} - \frac{a_2}{a_1(a_1+a_2U)}\, dU \\ &= \left.\frac{\ln\left|\frac{U}{a_1+a_2U}\right|}{a_1}\right|_{U=U_0}^{U=U^*\pm\varepsilon_{\text{exit}}} \\ T_f &= \frac{1}{a_1}\ln\left|\frac{(U^*\pm\varepsilon_{\text{exit}})(a_1+a_2U_0)}{U_0[a_1+a_2(U^*\pm\varepsilon_{\text{exit}})]}\right|. \end{aligned} \tag{S8}$$

If $a_2 = 0$ we have

$$T_f = \frac{1}{a_1}\ln\left(\frac{U^*\pm\varepsilon_{\text{exit}}}{U_0}\right), \tag{S9}$$

while if $a_1 = 0$ L'Hopital's rule gives

$$T_f = \frac{U^* - U_0 \pm \varepsilon_{\text{exit}}}{a_2 U_0 (U^* \pm \varepsilon_{\text{exit}})}. \tag{S10}$$

We note that the case $a_1 = 0$ is similar in nature to a center manifold reduction. Furthermore, some general observations, the final time is inversely proportional to the lowest order (nonzero) coefficient and diverges logarithmically in the slow manifold case, and harmonically in the center manifold case. These relations allow us to read off the time to an event in parameter regions where this approximation is suitable. Consider the approach to the fixed point $U^* = 0$ and suppose $a_n$ is the first nonzero coefficient of Eq. (S7), then we see that

$$T_f \approx \begin{cases} \frac{1-(\varepsilon_{\text{exit}}/U_0)^{n-1}}{|a_n|(n-1)\varepsilon_{\text{exit}}^{n-1}} & \text{if } n > 1, \\ \frac{1}{|a_1|}\ln\left(\frac{U_0}{\varepsilon_{\text{exit}}}\right) & \text{if } n = 1. \end{cases} \tag{S11}$$

This provides a useful tool to check the order of the slow manifold. By varying the size of $\varepsilon_{\text{exit}}$, we can numerically determine the scaling law to find the first dominant term in the slow manifold.

# 3 Slow manifold calculation

We calculate an approximation to the slow manifold explicitly in the case where free-riders win and $|c(ar-1)| \ll 1$. We calculate a Taylor expansion of the slow manifold in terms of $U$, where we assume $V = V(U)$ and $G = G(U)$ and we also expand in $\epsilon \ll 1$. At first order, we obtain

$$V(U) = 1 - cr + \left\{\frac{cr[1+\lambda'(0)]-1}{1+c(ar-r-1)} + \frac{c^2r(ar-1)\lambda'(0)}{(cr-1)[1+c(ar-r-1)]}\epsilon\right\}U + O(\epsilon^2, U^2), \tag{S12}$$

$$G(U) = \left[\frac{1}{1-cr} + \frac{c(1-ar)}{(1-cr)^2}\epsilon\right]U + O(\epsilon^2, U^2). \tag{S13}$$

The expression to second order is more complicated, and with the aid of Mathematica, we obtain to second order

$$\dot{U} = c(ar-1)U + \left[\frac{cr\lambda'(0)}{1-cr} - \epsilon c^2 r(ar-1)\lambda'(0)\left(\frac{1-c(1+r)+a(2cr-1)}{(cr-1)^2[1+c(ar-r-1)]}\right) + a\left(-\frac{cr\lambda'(0)+cr-1}{1+c(ar-r-1)} - 1\right)\right]U^2. \tag{S14}$$

To leading order, the $\varepsilon$-extinction time is then given by

$$T_f = \frac{1}{c(1-ar)}\ln\left|\frac{U_0}{\varepsilon_{\text{exit}}}\right|. \tag{S15}$$

For more details and the calculations for the other parameter regimes and fixed points, see the Mathematica notebook in the "spatialPGG" repository at https://github.com/MathOnco/spatialPGG.

# 4 Calculating time to slow manifold/coexistence times

When cell death is negligible, $\mu_U = \mu_V = 0$ (thus $c = 0$ in the non-dimensional case), coexistence is possible. To see this with $c = 0$ we can divide Eq. (S1) by Eq. (S2) and arrive at

$$\frac{dU}{dV} = \frac{U}{V}\left(1 + \frac{a-1}{\lambda(G)}\right).$$

If we make the assumption that $G$ equilibrates rapidly, we make the approximation that $G \approx G_0 = U_0/(U_0 + V_0)$, (the initial value of the public good). This allows us to solve this equation for $U$ and arrive at

$$U = U_0\left(\frac{V}{V_0}\right)^{1+\eta}, \tag{S16}$$

with $\xi = \frac{a-1}{\lambda(G_0)}$. Inserting Eq. (S16) into Eq. (**??**), we have

$$\dot{V} = V\lambda(G_0)\left\{1 - V\left[1 + \frac{U_0}{V_0}\left(\frac{V}{V_0}\right)^{\xi}\right]\right\}. \tag{S17}$$

We also make the observation that with the range for $a \in [0, 0.25]$ we see that $|\xi| \leq 0.25$ and so we consider it as a small parameter. For $t \to \infty$, a steady state is given by the solution to

$$\frac{U_0}{V_0^{1+\xi}}V^{1+\xi} + V - 1 = 0.$$

Expanding $V$ in $\xi \ll 1$ we obtain

$$V^* \approx \frac{1}{1 + \frac{U_0}{V_0^{1+\xi}}}. \tag{S18}$$

# 5 Calculation of wavefront speed in 1D

Although traveling wave solutions can not exist indefinitely, they were still observed in our simulations of the invasion processes (free-riders and producers separated by a domain wall) as the primary mechanism to reach a homogeneous state.

Following the work in [4], we seek an approximation to the speed of the wavefront. The instability of the unstable state drives the dynamics. The procedure is as follows. First, we linearize about unstable state, the state that is invaded. We assume an Ansatz, that is of the form of a traveling wave $e^{\Lambda t - Kx} = e^{-K(x - \Lambda/Kt)}$. Here it is clear that $1/K$ is the length scale that determines the width of the transition zone and $\eta = \Lambda(K)/K$ is the speed of the wave. The wave travels at the minimum possible speed which is obtained by looking at the extremum $\frac{d\eta}{dK} = 0$.

## 5.1 Free-rider invasion

Suppose the producer-only steady state is unstable. Then we consider a perturbation about that state, $U = 1 - \frac{c}{\lambda(1)+a-1} + e^{\Lambda t - Kx}\tilde{U}$, $V = e^{\Lambda t - Kx}\tilde{V}$ and $G = 1 + e^{\Lambda t - Kx}\tilde{G}$, where the tilde variables are meant to be small perturbations. Plugging this into Eq. (S1)-(S3) and neglecting higher order terms in the tilde vaiables, we arrive at a linear system $\mathbf{A}(\Lambda, K)\mathbf{p} = \mathbf{0}$ where $\mathbf{p} = (\tilde{U}\ \tilde{V}\ \tilde{G})^T$. We are interested in nontrivial solutions to this linear equation, which implies we are looking for when the determinant is 0. This leads to three separate values for $\Lambda$,

$$\Lambda_1 = K^2\gamma_U + 1 - a + c - \lambda(1),\ \Lambda_2 = K^2\gamma_V - cr + \frac{c\lambda(1)}{\lambda(1) - 1 + a},\ \Lambda_3 = \frac{1}{\epsilon}\left[(K^2 - 1) + \frac{c}{\lambda(1) + a - 1}\right].$$

Since the producer-only state is unstable, it must be that one of the eigenvalues is positive and the relevant eigenvalue is $\Lambda_2$, Since we are interested in the speed of the front, we are looking at

$$\eta_2 := \frac{\Lambda_2}{K} = \gamma_V K + \frac{c[\lambda(1) - r(\lambda(1) + a - 1)]}{K(\lambda(1) + a - 1)}.$$

Minimizing over $K$, we arrive at

$$|\eta_2| = 2\sqrt{c\gamma_V \left[\frac{\lambda(1)}{\lambda(1) + a - 1} - r\right]}. \tag{S19}$$

## 5.2 Producer invasion

If instead, we linearize about the free-rider state, we use $U = e^{\Lambda t - Kx}\tilde{U}$, $V = 1 - cr + e^{\Lambda t - Kx}\tilde{V}$ and $G = e^{\Lambda t - Kx}\tilde{G}$. In an analogous manner to the producer state, we arrive at

$$\Lambda_1 = K^2\gamma_U + c(ar - 1),\ \Lambda_2 = K^2\gamma_V - (1 - cr),\ \Lambda_3 = \frac{K^2 - (1 - cr)}{\epsilon}$$

Since the free-rider only state is unstable, it must be that $ar - 1 > 0$ and so the relevant eigenvalue is $\Lambda_1$,

$$\eta_1 := \frac{\Lambda_1}{K} = \gamma_U K + \frac{c(ar - 1)}{K}.$$

Minimizing over $K$, we arrive at

$$|\eta_1| = 2\sqrt{c\gamma_U(ar - 1)}. \tag{S20}$$

An important side note for the invasion of producers into the domain is in the case that $1 - cr < 0$. In this case, the free-rider only state is not only unstable, but is biologically infeasible and so the wave speed calculation will be inaccurate since the trajectory in space is no longer traveling from that point to the producer-only state. Instead, it is traveling from the mass-extinction state towards the producer state. The corresponding eigenvalues $\Lambda$ can be found in an analogous manner and we obtain the estimate for the traveling wave speed,

$$|\eta| = 2\sqrt{\gamma_U[\lambda(G^*) + a - 1 - c]}. \tag{S21}$$

## 5.3 Wavefront speed in higher dimensions

The wave front speed in higher dimensions can be calculated provided that the wave front is strictly 1D. That is, if the wave is of the form $u(x, y, z, t) = u(x - \eta t)$, then the wave speed can be calculated and is analogous to the previous section (Table A). In principle, other wavefronts can be calculated, for example, the radially symmetric wave that starts from the center of the 3D domain, which invade outward. However, since the domain is finite, the boundaries play a significant role in affecting the speed and so we restrict our attention to 1D-traveling waves.

| **Length** | **$\varepsilon$-extinction time (1D, 2D, 3D)** |
|---|---|
| 10 | (196.69, 197.04, 197.04) |
| 20 | (209.97, 210.21, 210.21) |
| 40 | (235.01, 235.18, 235.18) |

**Table A: Comparison of 1D-plane waves in higher dimensions.** We observe similar times regardless of the dimensionality of the domain.

# 6 Calculating $\varepsilon$-extinction times using a traveling wave ansatz

Consider the general class of Fisher models

$$\vec{U}_t = \mathbf{D}\vec{U}_{xx} + \mathbf{R}\vec{F}(\vec{U}), \tag{S22}$$

where $\mathbf{D}, \mathbf{R} \in \mathbb{R}^{n\times n}$ are diagonal matrices containing the diffusion coefficients and growth rates respectively. We introduce two characteristic scales, to be chosen, $x/L_x = X$ and $\tau = t/T$, which leads to

$$\frac{1}{T}\vec{U}_\tau = \frac{\mathbf{D}}{L_x^2}\vec{U}_{XX} + \mathbf{R}\vec{F}(\vec{U}). \tag{S23}$$

Define $r = \max \mathbf{R}$ and $D = \max \mathbf{D}$. We will manipulate these different scales to analyze the system. First, suppose that $L_x \gg \sqrt{D/r}$

$$\frac{1}{T}\vec{U}_\tau = \frac{\mathbf{D}}{L_x^2}\vec{U}_{XX} + \mathbf{R}\vec{F}(\vec{U}). \tag{S24}$$

This implies that spatial variations can be neglected at large length scales. To balance the remaining two terms, we set $T = 1/r$ and see that

$$\vec{U}_\tau = \frac{1}{r}\mathbf{R}\vec{F}(\vec{U}). \tag{S25}$$

This observation implies that the system evolves independently over a large spatial scale and is governed solely by the growth term. Hence after rescaling time back we see that $\vec{U}_t = \mathbf{R}\vec{F}(\vec{U})$ and so the time for equilibration at this scale is given by $\tau_{\mathrm{ODE}}(\vec{U}_0)$, where $\vec{U}_0$ is the initial data.

Suppose instead that $L_x \ll \sqrt{D/r}$. To balance we set $T = L_x^2/D$. and require that $T \ll r$. Hence in this regime, we see

$$U_\tau = U_{XX}, \tag{S26}$$

which implies that at small spatial scales, variations are damped out exponentially quickly on the order of $T \ll 1/r$.

Moving on to the last case, we suppose that $L_x = \sqrt{D/r}$. Setting $T = 1/r$ leads to all three terms balancing. At this point, we look for traveling wave solutions of the form $\vec{U}(X,\tau) = \vec{U}(X - \eta\tau) = \vec{U}(z)$. Inserting this into equation (S23) leads to

$$0 = \frac{\mathbf{D}}{D}\vec{U}'' + \eta\vec{U}' + \frac{1}{r}\mathbf{R}\vec{F}(\vec{U}). \tag{S27}$$

We observe that the speed of this wave if it exists should have the following characteristic scale $\eta \sim L_x/T = \sqrt{rD}$. Then the time it would take for this wave to travel a distance $d = L/2$ (half the domain) is given by

$$\tau_{\text{wave propagation}} \sim \frac{d}{\eta} = \frac{L}{2\sqrt{rD}}. \tag{S28}$$

In our case though, the domain is finite and so this wave will eventually reach the boundary. Will the wave just pass through? No. It is clear that in the case of a no-flux boundary, we require $u_x(L,t) = U(L - \eta t) = 0$ for all $t$, which clearly cannot be satisfied once the wave has reached the boundary. What then is the mechanism to finish equilibration once the traveling wave reaches the boundary? To answer this, we note that it is clear that $\tau$ should be a continuous function of $L$. Furthermore, provided that the wave speed is finite, we expect $\tau_{\text{wave propagation}}(L)$ to be a linear function of $L$. Recall also that for $L$ small, we are smaller than the transition width of the traveling wave, and are in the region where spatial variations are smoothed rapidly $T \ll 1/r$, hence we can replace $\vec{U}$ by its

spatially averaged value $\vec{u}$. To see this, consider the average value by integrating over the domain

$$\begin{aligned}\frac{1}{L}\int_0^L \vec{U}_t \; dx &= \frac{1}{L}\int_0^L \mathbf{D}\vec{U}_{xx} + \mathbf{R}\vec{F}(\vec{U}) \; dx \\ \vec{u}_t &= \frac{\mathbf{R}}{L}\int_0^L \vec{F}(\vec{U}) \; dx \\ \vec{u}_t &= \frac{\mathbf{R}}{L}\int_0^L \vec{F}(\vec{u}) + L\vec{\phi}\frac{\partial \vec{F}}{\partial \vec{U}}\bigg|_{\vec{U}=\vec{u}} \; dx \\ \vec{u}_t &= \mathbf{R}\vec{F}(\vec{u}) + \mathbf{R}\frac{\partial \vec{F}}{\partial \vec{U}}\bigg|_{\vec{U}=\vec{u}}\int_0^L \vec{\phi} \; dx \\ \vec{u}_t &= \mathbf{R}\vec{F}(\vec{u}) + \mathcal{O}(L).\end{aligned}$$

The last part follows where we have defined $\vec{U} = \vec{u} + L\vec{\phi}$ and the fact that spatial variations are removed quickly. Thus, as L $\rightarrow 0$ it is clear that

$$\lim_{L\rightarrow 0} \tau = \tau_{\text{ODE}}(\vec{u}). \tag{S29}$$

By continuity then

$$\tau = \tau_{\text{ODE}}(\vec{u}) + \tau_{\text{wave propagation}}(L) + \tau_{\text{wave formation}}. \tag{S30}$$

It is clear that the last term should tend to 0 if we started with an initial condition which was close to the form of the wave. Despite using a step function initial condition, this time to formation was typically much smaller than the first two terms. We also note that the third term is independent of length provided the domain is sufficiently large enough for wave formation. This allows us to easily calculate numerically the traveling wave speed (see main text for details).

We have shown that we may approximate the total time by this superposition of terms and that the initial data for the ODE term is given by the spatially averaged value of the wave profile, but what is this value? To answer this, we first assume that there is one state $\vec{U}^*$ that is invading the state $\vec{V}^*$. First, we integrate equation (S22) by transferring into the co-moving reference frame $z = x - \eta t$, the equation for the traveling wave profile from $-\xi$ to $\xi$ in such a way that $\vec{U}(-\xi) = \vec{U}^*, \vec{U}(\xi) = \vec{V}^*$. We will also define for notational simplicity the difference $\vec{w} := U^* - V^*$ and the average value $\vec{\mu} = (U^* + V^*)/2$. We note that we are actually integrating element-wise and so all vectors appearing in multiplication (or division) are meant to be done element-wise.

$$\int_{-\xi}^{\xi} \mathbf{D}\vec{U}'' + \eta\vec{U}' + \mathbf{R}\vec{F}(\vec{U}) \; dz = 0 \tag{S31}$$

$$\begin{aligned}\eta[\vec{U}(\xi) - \vec{U}(-\xi)] + \int_{-\xi}^{\xi} \mathbf{R}\vec{F}(\vec{U}) \; dz &= 0 \\ \int_{-\xi}^{\xi} \mathbf{R}\vec{F}(\vec{U}) \; dz &= \eta(\vec{U}^* - \vec{V}^*)\end{aligned}$$

$$\frac{1}{\eta\vec{w}}\int_{-\xi}^{\xi} \mathbf{R}\vec{F}(\vec{U}) \; dx = \vec{1}. \tag{S32}$$

We will make use of the following identities

$$\int_{-\xi}^{\xi} \vec{U}\vec{U}' \; dz = -\vec{w}\vec{\mu}, \tag{S33}$$

$$\int_{-\xi}^{\xi} \vec{U}'\vec{U}'' \; dz = \vec{0}. \tag{S34}$$

Next, we multiply by $\vec{U}$ and integrate equation (S27) to obtain

$$\int_{-\xi}^{\xi} \vec{U}\mathbf{R}\vec{F}(\vec{U})\ dz = \eta\vec{w}\vec{\mu} + \int_{-\xi}^{\xi} \mathbf{D}(\vec{U}')^2\ dz. \tag{S35}$$

At this point, we make the observation that equation (S32) is a probability distribution. Hence equation (S35) can be formalized as

$$\langle\vec{U}\rangle = \frac{1}{\eta\vec{w}}\int_{-\xi}^{\xi} \vec{U}\mathbf{R}\vec{F}(\vec{U})\ dz = \vec{\mu} + \frac{\mathbf{D}}{\eta\vec{w}}\int_{-\xi}^{\xi} (\vec{U}')^2\ dz. \tag{S36}$$

Where $\langle\cdot\rangle$ signifies the average value. We can further refine this by considering once more equation (S31) by multiplying by $U'$ and integrating over space to obtain

$$\frac{1}{\vec{w}}\int_{-\xi}^{\xi} (\vec{U}')^2\ dz = \frac{1}{\eta\vec{w}}\int_{-\xi}^{\xi} \vec{U}'\mathbf{R}\vec{F}(\vec{U})\ dz = -\langle\vec{U}'\rangle. \tag{S37}$$

Inserting this expression into equation (S36), we obtain

$$\langle\vec{U}\rangle = \vec{\mu} - \frac{\mathbf{D}\langle\vec{U}'\rangle}{\eta}. \tag{S38}$$

Thus for $\mathbf{D}/\eta \ll 1$, we may approximate the mean of $\vec{U}$ as simply the average value between the two states.

# 7 Calculation of time to coexistence in the absence of cell death

In the case $c = 0$ (no death), the ODE model gives an approximate time for coexistence to the line $U + V = 1$. Simulations of the spatial model show a similar rapid approach to the line with the *average* population. However, the system continues to run till it is homogeneous. Since there is no traveling wave in this scenario, the only method to reach homogeneity is that of diffusion. In a similar way to the wavefront problem, we assume that the total time is given by

$$\tau_{\text{total}} = \tau_{\text{coexistence}} + \tau_{\text{diffusion}}. \tag{S39}$$

We use the approximation that $U + V = 1$ for calculating the diffusion time. This uncouples $U$ and $V$ and reduces Eq. (S1)-(S3) to

$$\dot{U} = \gamma_U \nabla^2 U, \tag{S40}$$

$$\dot{V} = \gamma_V \nabla^2 V, \tag{S41}$$

$$\epsilon\dot{G} = \nabla^2 G + U - G. \tag{S42}$$

We expand the solutions of $U, V, G$ using a cosine series $U,\ V,\ G \sim \sum a_n(t)\cos(\omega_n x)$, where $\omega_n = \frac{n\pi}{L}$. The solution for $U$ is given by

$$U = \sum_{n=0}^{\infty} U_n e^{-\gamma_U \omega_n^2 t}\cos(\omega_n x),$$

where

$$U_n = \begin{cases} \frac{2}{L}\int_0^L U^0(x)\cos(\omega_n x)\ dx & \text{if } n \neq 0, \\ \frac{1}{L}\int_0^L U^0(x)\ dx & \text{if } n = 0, \end{cases}$$

and $U^0(x) := U(x, 0)$. The solution for $V$ is analogous and we note that the time for $G$ to equilibrate is dependent on $U$ and the time is exponentially small.

As an example, we choose the domain wall initial conditions $U = I_u H(L/2 - x)$ and $V = I_v H(x - L/2)$. We obtain

$$U = \frac{I_u}{2} + \frac{2I_u}{\pi} \sum_{n=0}^{\infty} \frac{(-1)^n \exp\left(-\gamma_U \omega_{2n+1}^2 t\right)}{2n+1} \cos(\omega_{2n+1} x),$$
$$V = \frac{I_v}{2} - \frac{2I_v}{\pi} \sum_{n=0}^{\infty} \frac{(-1)^n \exp\left(-\gamma_V \omega_{2n+1}^2 t\right)}{2n+1} \cos(\omega_{2n+1} x).$$

The exit criterion is satisfied when $\|U_t\|_1 < \varepsilon_{\text{exit}}$ and $\|V_t\|_1 < \varepsilon_{\text{exit}}$. Noting that $n = 1$ governs the time to homogeneity ($n > 1$ is subdominant), we obtain

$$\tau_{\text{diffusion}} = \frac{L^2}{\gamma_U \pi^2} \ln\left(\frac{4\gamma_U I_u}{\delta L^2}\right). \tag{S43}$$

A similar result is obtained in higher dimensions. The procedure is the same for any initial condition. However, we note that the choice of the exit criterion will change the form of $\tau_{\text{diffusion}}$. Examples include $\|U_t\|_\infty$ or $\|U_x\|_p$ (the p-norm). For example, using $\|U_x\|_1$ we obtain

$$\tilde{\tau}_{\text{diffusion}} = \frac{L^2}{\gamma_U \pi^2} \ln\left(\frac{4U_u}{\delta \pi L}\right). \tag{S44}$$

It is easy to see that this choice leads to a larger increase for increasing length.

# 8 Ruling out pattern formations

We have solved Equations. (S1)-(S3) with different initial conditions in one-,two- and three-dimensional systems. Though the time to $\varepsilon$-extinction or coexistence can vary, all systems eventually reached spatial homogeneity. A main cause of spatial pattern formations in diffusive systems is through diffusion-driven instability (DDI) which occurs through a mechanism known as a Turing bifurcation. We show here that the model does not admit a DDI via a Turing bifurcation.

To begin, we recall that for DDI to occur, we require the homogeneous fixed state be linearly stable in the absence of diffusion. Stability in the absence of diffusion implies $\frac{1}{r} > \max(a, c)$. For DDI, we require the existence of a perturbation which drives at least one eigenvalue positive. This leads to the condition that either $-[c(1 - ar) + k^2\gamma_U] > 0$ or $-(1 - cr + k^2\gamma_V) > 0$. Clearly, neither condition can be satisfied and so no DDI is possible off the free-rider only state. The situation is analogous for the producer-only state and the extinction state, where the addition of diffusion only further drives the stability of the point and does not cause any instability.

The reasons for the markedly different behavior compared to that observed in [5, 6] is primarily due to their system mimicking the behavior of an activator-inhibitor system [7, 8], where cooperators play the role of activators and defectors - the role of inhibitors. These systems are well-known to exhibit DDIs. In contrast, our system is based on a competitive Lotka-Volterra model with a growth rate dependent on the amount of public good available, which is determined by the amount of producers.

# 9 Different initial conditions converge to the homogeneous solution

Our simulations show that systems with higher initial spatial order take longer to converge to the homogeneous solution, see Figure A. We also observe that even though the total distance the free-rider population must is invade is the same ($L/2$), the traveling waves in figure A2 occur simultaneously and shows that each wave is traveling $L/4$. Hence the wave front time should be roughly half. The well-mixed model takes 476.79 cell cycles to fixate. The difference between the two times gives the total time for the two-wave travel, which is 503.51. The speed of the wave is given by Eq. (S19) and is equal to 0.1126. Hence the two waves traveled approximately 56.69, a slight overestimate as it should be 50. The discrepancy is likely due to the interaction of the left and right traveling wave

fronts being in close proximity at the beginning. In Figure A3, we show the difference between the mean population trajectory of the traveling wave (both waves travel the same mean path) as compared to the well-mixed (ODE) model, which is the orange, dashed line. As expected the trajectories are not the same in this case (compare with Fig. 1, Main Text) as this is due to the traveling wave following the heteroclinic connection from the stable state into the unstable state [9]. However, note that as we approach the fixed point, we converge to the same path the well-mixed model is taking. This is in agreement with the argument presented in Appendix 6.

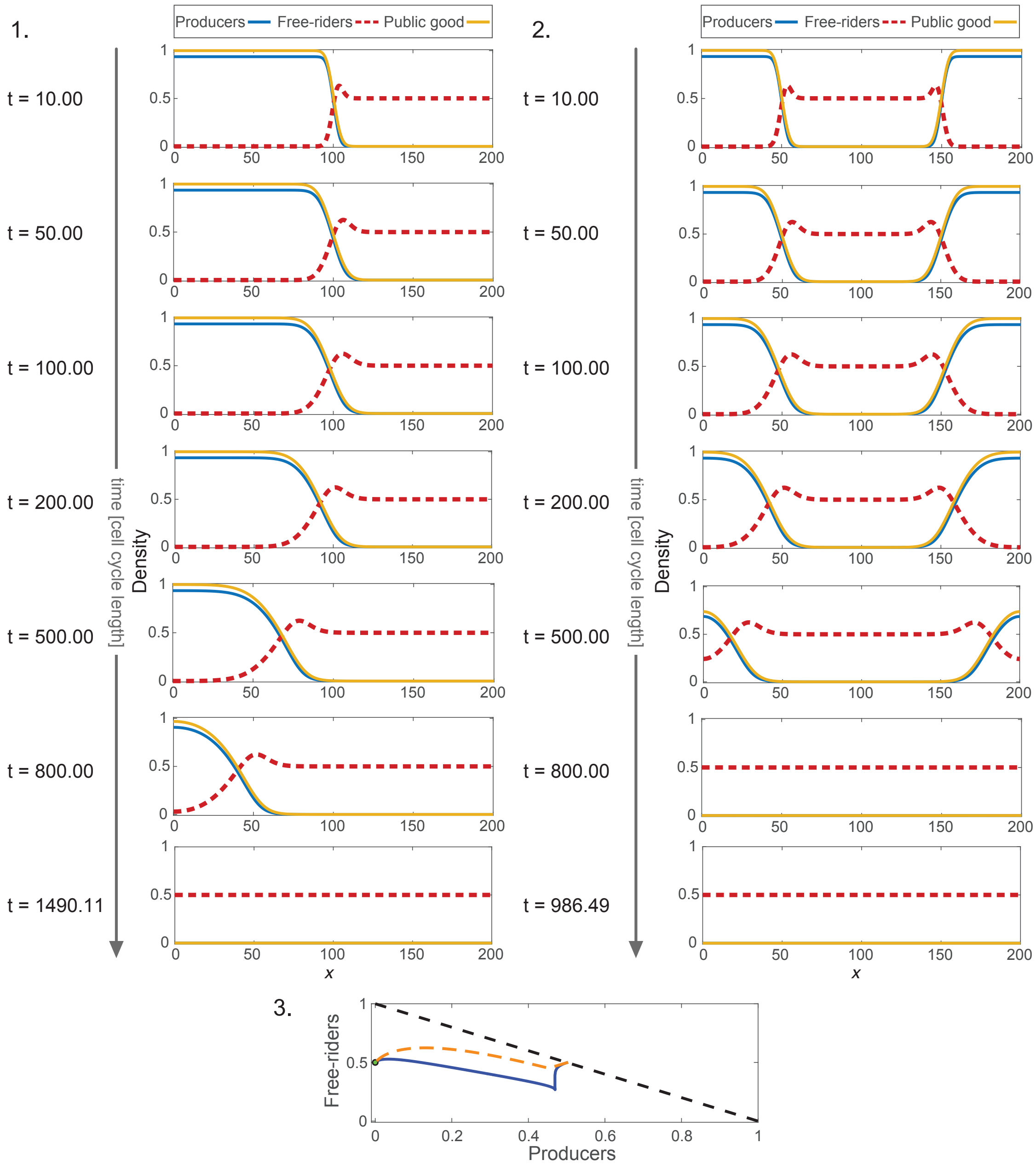


**Figure A: More spatially ordered systems take longer to converge to the homogeneous solution.** **1**: The domain wall initial condition. A single traveling wave is observed. **2**: The centered domain wall. Two traveling waves in opposite directions are observed. **3**: The trajectory in mean producer and free-rider space. The orange, dashed curve is the slow manifold predicted by the non-spatial model. As expected the spatial average travels a different manifold until it gets close to the fixed point. Other (non-dimensional) parameters used in all panels: $a = 0.9,\ \beta = 5,\ \sigma = 2\ r = 1,\ c = 0.5,\ \gamma_U = \gamma_V = 0.5,\ \epsilon = 2 \times 10^{-3}$.

## 10 Uncorrelated initial conditions remove domain-length dependence

To show that uncorrelated initial conditions lead to non-length dependent $\varepsilon$-extinction times, we simulated $L = 50, 100, 200, 400$ and computed the Moran $I$ statistic with weighting matrix $w = \delta_{i,i-1} + \delta_{i,i+1}$. The statistic ranges from -1 (perfectly dispersed) to 1 (perfectly separated, and 0 (random). Let $y$ be some spatial data and $\bar{y}$ be the mean, then the Moran statistic is given by

$$I = \frac{N}{W} \frac{(y-\bar{y})^T w (y-\bar{y})}{(y-\bar{y})^T (y-\bar{y})}, \tag{S45}$$

where $N$ is the number of grid points and $W = \sum_i \sum_j w_{ij} = 2(N-1)$. If there is no spatial correlation, the expected value of this statistic is $E[I] = -(N-1)^{-1}$. We tested this by taking the mean of 50 simulations in 1D with different $L = 50, 100, 200, 400$ and the results are summarized in table B.

| **Length** | **Moran I** | **$\varepsilon$-extinction time** |
|---|---|---|
| 50 | -0.0212 | 481.18 |
| 100 | -0.003 | 480.36 |
| 200 | -0.0086 | 479.31 |
| 400 | -0.0067 | 480.33 |

**Table B: Moran $I$ statistic measures spatial correlation.** Uncorrelated initial conditions as measured by the Moran $I$ statistic, which shows the length of the domain does not impact the time to $\epsilon$-extinction when uncorrelated ICs are used.

# References

1. Archetti M, Ferraro DA, Christofori G. Heterogeneity for IGF-II production maintained by public goods dynamics in neuroendocrine pancreatic cancer. Proceedings of the National Academy of Sciences USA. 2015;112:1833–1838.

2. Rabkin R, Fervenza FC, Maidment H, Ike J, Hintz R, Liu F, et al. Pharmacokinetics of insulin-like growth factor-1 in advanced chronic renal failure. Kidney International. 1996;49(4):1134–1140.

3. Kimmel GJ, Gerlee P, Brown JS, Altrock PM. Neighborhood size-effects shape growing population dynamics in evolutionary public goods games. Communications Biology. 2019;2(53):1–10.

4. Bayliss A, Volpert V. Complex predator invasion waves in a Holling–Tanner model with nonlocal prey interaction. Physica D. 2017;346:37–58.

5. Wakano JY, Nowak MA, Hauert C. Spatial dynamics of ecological public goods. Proceedings of the National Academy of Sciences USA. 2009;106:7910–7914.

6. Wakano JY, Hauert C. Pattern formation and chaos in spatial ecological public goods games. Journal of Theoretical Biology. 2011;268(1):30–38.

7. Satnoianu RA, Menzinger M, Maini PK. Turing instabilities in general systems. Journal of Mathematical Biology. 2000;41(6):493–512.

8. Silva FdS, Viana R, Lopes S. Pattern formation and Turing instability in an activator–inhibitor system with power-law coupling. Physica A: Statistical Mechanics and its Applications. 2015;419:487–497.

9. Kolmogorov A, Petrovskii I, Piskunov N. Study of a diffusion equation that is related to the growth of a quality of matter, and its application to a biological problem. Byul Mosk Gos Univ Ser A Mat Mekh. 1937;1(1):26.
